# Precision phage therapy in the AI/ML era: a systematic review of discovery-to-clinical translation evidence

**DOI:** 10.3389/fmicb.2026.1873220

**Published:** 2026-09-09

**Authors:** Najwa Menwer Alharbi

**Affiliations:** Department of Biological Sciences, Faculty of Science, King Abdulaziz University, Jeddah, Saudi Arabia

**Keywords:** antimicrobial resistance, artificial intelligence, clinical translation, foundation models, genome annotation, graph neural networks, host–phage interaction, machine learning

## Abstract

In precision phage therapy, artificial intelligence and machine learning (AI/ML) is being applied for phage detection, genomes/proteome annotation, host prediction, resistance profiling and therapeutic optimization. Yet the landscape remains fragmented owing to a lack of systematic synthesis of its translational maturity, methodological robustness and pipeline coverage. We performed a systematic review of AI/ML tools relevant to precision phage therapy. Web of Science, PubMed, MEDLINE, Scopus and IEEE Xplore were used to identify records. Of 6,969 identified records, 2,214 duplicates were removed, leaving 4,755 unique records for title/abstract screening; 510 full-text articles were assessed for eligibility, and 128 studies were included in the final synthesis. Studies were classified across a nine-module precision phage therapy pipeline by architectural family, validation maturity, accessibility, translational readiness (AI_PTRL), and methodological quality using an adapted PROBAST framework. The 128 included studies were published between 2012 and 2025, with a median publication year of 2023 and peak activity during 2022–2025. The large majority were methodological/tool development studies (115/128, 89.8%) and these predominantly described *in silico* validation (101/128, 78.9%). The pipeline coverage was uneven, with the highest concentration in the host range and interaction determinants (*M*4, 43/128, 33.6%) and genome/protein annotation and functional inference (*M*3, 33/128, 25.8%). Safety screening, therapeutic design, and infrastructure modules were comparatively underrepresented. From an architectural standpoint, hybrid/integrated systems (HIS) (39/128, 30.5%) and classical machine learning (CML) (38/128, 29.7%) were the most common approaches with substantial diversification after 2022. The overall translational readiness was modest (median AI_PTRL: 6, IQR 4–6). Methodological assessment demonstrated high evaluation risk of bias (104/128, 81.2%) and substantial development concern (88/128, 68.8%), especially in Domain 4 (analysis). AI/ML for precision phage therapy is a rapidly growing and diversifying field, particularly in host–phage interaction prediction, however efforts are still limited for downstream precise phage therapy tasks. Experimental validation and real-world deployment remain limited with methodological limitations due to analytical design and evaluative approaches. Future work should prioritize rigorous validation frameworks, higher-quality and more complete representative datasets, stronger safety screening, therapeutic optimization, and clinically actionable decision support.

## 1 Introduction

Antimicrobial resistance (AMR) has emerged as one of the major global health threats, with an estimate that by 2050 it will directly account for up to 10 million deaths per year and have a profound human and economic impact (Walsh et al., 2023; Naghavi et al., 2024). This crisis is driven by a combination of the inappropriate and excessive use of antibiotics in human health, agriculture and animal husbandry when paired with reduced novel antibacterial agent discovery (Walsh et al., 2023). Bacteriophage (phage) therapy is experiencing a revival as an alternative treatment modality. Phages are bacterial viruses with high specificity that can kill target pathogens and spare the surrounding microbiome (Salmond and Fineran, 2015; Adler et al., 2025). Because their mechanisms of action differ fundamentally from those of antibiotics, they remain a potentially effective alternative against multidrug-resistant (MDR), extensively drug-resistant (XDR), and pan-drug-resistant infections (PDR) (Gordillo Altamirano and Barr, 2019). The modern ambition is therefore not limited to empirical or exclusively personalized phage application. Precision phage therapy encompasses data-driven selection of natural phages, rational formulation of phage combinations, and, where appropriate, engineering of phages or therapeutic components according to the infecting bacterial isolate, host context, and predicted treatment dynamics (Pirnay et al., 2018; Hatfull et al., 2022). Alongside personalized approaches, recent work has demonstrated strategies for developing broadly effective phage–antibiotic cocktails, suggesting that future phage therapeutics may encompass both individualized and broader-spectrum formulations, depending on the clinical context and therapeutic objective (Kim et al., 2024b). Clinical experience has increasingly expanded beyond individual case reports, with personalized bacteriophage therapy being evaluated across specialized clinical programs and larger real-world cohorts. Schooley et al. (2017), for example, reported the development and administration of personalized phage cocktails for a patient with disseminated multidrug-resistant *Acinetobacter baumannii* infection, including iterative reformulation in response to emerging phage resistance (Schooley et al., 2017). Subsequent experience included a US specialized phage-therapy program, which reported the first 10 consecutive cases of intravenous bacteriophage therapy for multidrug-resistant infections and highlighted practical considerations related to phage susceptibility testing, regulatory pathways, phage resistance, and concomitant antibiotic use (Aslam et al., 2020). More recently, a multicentre, multinational retrospective analysis of 100 consecutive personalized phage-therapy cases reported clinical improvement in 77.2% of infections and bacterial eradication in 61.3%, although the uncontrolled observational design limits causal interpretation (Pirnay et al., 2024). In parallel, engineering bacteriophages for the treatment of a patient with MDR *Mycobacterium abscessus* has further expanded the therapeutic possibilities of phage-based interventions (Dedrick et al., 2019).

However, the path toward precision phage therapy is still limited by significant scientific and translational barriers. The first hurdle is therefore not necessarily phage availability, given the growing number of international phage collections and biobanks, but rather discovery and matching: identifying and screening available phages for effective activity against a specific clinically relevant bacterial isolate (Iredell et al., 2024; Resch et al., 2024). This remains challenging because phages often exhibit narrow or strain-specific host ranges and experimentally confirmed activity against a target isolate cannot be reliably inferred from species-level identity or sequence information alone (Hyman, 2019). This challenge has created a strong need for computational approaches that can prioritize candidate phages and predict phage–host compatibility before experimental testing. The second barrier is the genomic annotation gap. A substantial proportion of phage genes remain hypothetical or poorly characterized, limiting confident assignment of protein function and biological role. This annotation gap complicates interpretation of phage genomes and can constrain the reliable inference of biologically and therapeutically relevant properties from sequence data, thereby motivating the development of computational approaches capable of inferring functional information beyond conventional homology-based annotation (Grigson et al., 2023, 2025; Culot et al., 2025). The uncertainty is further increased by bacterial anti-phage defense systems, including restriction-modification, abortive infection, and CRISPR-Cas mechanisms, which can interfere with phage replication following infection, as well as by phage-resistance mechanisms such as receptor modification or alteration that can prevent or reduce phage adsorption and entry (Labrie et al., 2010; Georjon and Bernheim, 2023). These mechanisms can substantially hinder therapeutic efficacy and contribute to the emergence of phage resistance during treatment (Hampton et al., 2020).

The clinical application of generalized or personalized phage therapy remains constrained by several pharmacological and translational challenges (Faltus, 2024; Turner et al., 2024). Unlike conventional small-molecule antibiotics, phages are self-replicating biological entities whose pharmacokinetic and pharmacodynamic behavior is influenced by factors such as bacterial density, phage–host interactions, immune clearance, and tissue distribution, complicating the development of conventional PK/PD frameworks (Dabrowska and Abedon, 2019; Nilsson, 2019; Nang et al., 2023). Dosing and persistence therefore remain difficult to predict, particularly because therapeutic exposure may vary according to the route of administration, infection site, bacterial burden, and host immune response (Suh et al., 2022). Manufacturing and formulation present additional challenges, including achieving reproducible phage titres, adequate purification, stability, sterility, and removal of bacterial contaminants such as endotoxin/LPS (Pirnay et al., 2015; Hietala et al., 2019; Bretaudeau et al., 2020). Finally, regulatory pathways remain heterogeneous across jurisdictions, reflecting the biological complexity of phages, their capacity for evolution, and the need to accommodate both standardized and patient-specific preparations (Verbeken and Pirnay, 2022; Yang et al., 2023).

Collectively, these barriers suggest that precision phage therapy is not just a microbiological or clinical challenge but also an informatics and systems level one, demanding the integration of large, heterogeneous and high-dimensional datasets. The power of artificial intelligence and machine learning (AI/ML) in this context is that these methods offer the ability to transform complex biological datasets into predictive models. AI/ML methods are being explored along the entire phage therapeutic pipeline: from genome decoding and functional annotation, to host prediction, defense-system profiling, and optimization of therapeutics. Sequence-based characterization of phage proteins and lifestyle states has been increasingly advanced using deep learning (DL) and foundation-model approaches, as represented by tools including virionfinder, PhaVIP, and PhaTYP (Fang and Zhou, 2021; Shang et al., 2023c,b). Similarly, phage–host interaction prediction has advanced with graph-based, hybrid and ensemble models like CHERRY (Shang and Sun, 2022), GSPHI (Pan et al., 2023a), GERMAN-PHI (Wang et al., 2023), iPHoP (Roux et al., 2023), and DeepHost (Ruohan et al., 2022). AI has also started to assist in resistance-aware therapy design with anti-CRISPR and defense-system predictor tools like AcrNET (Li et al., 2023), PaCRISPR (Wang et al., 2020), and Deepdefense (Hauns et al., 2024), while downstream applications (i.e., cocktail optimization and data-guided dosing) are being pursued via frameworks such as PERPHECT for host-range engineering (Ataee et al., 2022) and ParetoPairFinder for dosing/time optimization framework (Plunder et al., 2024). Together, these advances demonstrate the growing potential of AI/ML to transform phage therapy from predominantly empirical screening toward more systematic and predictive decision support. Translating these computational capabilities into clinically actionable tools, however, will require further experimental validation, integration across the therapeutic workflow, and assessment of clinical and translational utility (Doud et al., 2025).

However, the fast-moving nature of this area has led to fragmentation as well. Phage biology AI/ML tools have mostly been built independently from one another, within rather narrow use-case definitions, while the larger architecture of the field has yet to be synthesized adequately. While there have been non-systems reviews of the potential of using AI in phage-related research (Mutalik and Arkin, 2022; Jung et al., 2024), none provides a systematic and pipeline-oriented inventory of how well-developed these tools are across the translational workflow, how mature their validation is, as well as whether they are available for researchers, or whether they span the gap toward meaningful clinical translation. As a result, researchers and clinicians still do not have a clear roadmap of methodological effort in this space, what domains are well-developed, where further development efforts may be justified, and where the substantive translational gaps remain.

The objective of this systematic review is to create a detailed evidence map of the AI/ML tools applicable in precision phage therapy. It aims to classify and describe some of the tools along a nine-module therapeutic pipeline, also in reference to time and geography as well as predominant AI/ML architectures used, maturities in different stages of validation and translation into clinical use and methodological robustness as well as accessibility. Through synthesizing across these frameworks, the review provides a foundation for recognizing where current emphases contribute toward sustained bottlenecks in advancing AI-enabled precision phage therapy from proof-of-concept to clinical practice and directs emergent energies toward bridging such divides.

## 2 Methods

### 2.1 Study design and review scope

This study serves as a systematic review of AI and machine learning (AI/ML) tools specifically directed toward studies related to precision phage therapy. To guide the evidence synthesis, we mapped all eligible tools against an end-to-end precision phage therapy pipeline and classified tools within a module-based taxonomy applied throughout screening, extraction and synthesis. We envisioned this framework to encompass the functional diversity of AI/ML applications across phage/virus detection and classification, safety and contamination screening, genome/protein annotation and functional inference, host range and interaction determinants, lifestyle and lysogeny, resistance and defense systems, therapeutic component discovery; therapeutic design (including *in silico* docking) and optimization and infrastructure/platform resources (detailed modules and functional categories are described in Supplementary Table S1).

### 2.2 Study selection

A systematic literature search was performed, according to PRISMA 2020 reporting guidance and PRISMA-S recommendations for literature-search reporting (Page et al., 2021), across five bibliographic databases: Web of Science, PubMed, MEDLINE, Scopus, and IEEE Xplore, between 2012 and 2025. Search framework, objective, information sources, and core Boolean structure and database-specific search strings used for literature retrieval details are shown in Supplementary Table S2.1, S2.2, respectively. The search strategy was designed to identify studies describing AI/ML methods, tools, platforms, or resources related to precision phage therapy endpoints. Retrieved records were exported for de-duplication and screening. Screening and all downstream including tool taxonomy and functional mapping, AI_PTRL determination and PROBAST + AI were assisted by three independent reviewers for accuracy.

### 2.3 Eligibility criteria

Included studies were required to report an explicit AI/ML component applied to a problem pertinent to the precision phage therapy pipeline. Eligible studies included primary reports of a tool or model that incorporated the use of AI/ML, as well as benchmarking studies, those describing a platform/database/resource or translational work and decision-support studies when AI/ML formed a substantive methodological input. We excluded studies with no explicit AI/ML method, conventional computational/bioinformatics work not using AI/ML methods, wet-lab or clinical phage studies without an AI/ML component, topics on phage or virome that did not fall within the defined scope of precision phage therapy, non-primary article types e.g., reviews and editorials or material unrelated phage research. The most frequent reasons for full-text exclusion were absence of an explicit AI/ML component, computational/bioinformatics studies without AI/ML, and wet-lab/clinical phage studies without AI/ML.

### 2.4 Data extraction

For each included study, data were extracted into a structured review dataset. Extracted fields included bibliographic information, publication year, country or countries of contribution, accessibility/availability mode, AI/ML methodological family, validation maturity, translational readiness, functional module assignment, and finer functional-category mapping where applicable. Unnamed tools were retained in the synthesis and coded as standardized unidentified entries when necessary to preserve the completeness of the evidence base. Study-level mappings of tool identity, module, functional category, and overall, AI/ML class were compiled into the master extraction Supplementary Workbook S3.1 used for quantitative synthesis and figure generation.

### 2.5 Functional taxonomy and architectural classification

To support landscape-level synthesis, included tools were mapped to an author predefined module-based precision phage therapy taxonomy. This allowed classification of tools according to their principal functional role within the therapeutic pipeline. In parallel, each study was assigned to an AI/ML architectural family, including classical machine learning (CML), deep learning (DL), hybrid/integrated systems (HIS), graph and geometric learning (GGL), foundation models (FM), probabilistic/statistical models (PSM), and generative models/design approaches (GM; Supplementary Workbook S3.1). These classifications were used to characterize the evolving methodological landscape and to examine how different architectures distributed across functional modules and over time.

### 2.6 Validation maturity and translational readiness assessment

Validation maturity was extracted and categorized according to the deepest level of evidence reported in each study: *in silico* only, *in silico* + *in vitro, in silico* + *in vivo*, or *in silico* + *in vitro* + *in vivo*. In addition, we developed an AI Phage Translational Readiness Level (AI_PTRL) framework, guided by the NASA Technology Readiness Level (TRL) framework (Technology Readiness Levels—NASA, 2023), to assess the translational maturity of AI/ML tools in precision phage therapy (Supplementary Workbook S3.1). The framework was conceptually adapted from NASA TRL logic but redefined to represent the progression of computational phage tools from biological rationale and model conception through prototype implementation, analytical validation, biological validation, preclinical testing, and clinical or real-world implementation.

Studies were assigned a readiness level from AI_PTRL 1 to AI_PTRL 9 (Supplementary Workbook S3.3) according to the highest stage achieved, provided that all preceding stages were satisfied. Internal validation corresponded to analytical testing within the original development setting, whereas external validation required evaluation using an independent dataset or benchmark. A separate generalizability stage captured robustness across biologically meaningful distribution shifts, such as previously unseen host taxa or phage families. *In vitro, in vivo*, and clinical or real-world deployment evidence were treated as progressively higher stages of translational maturity (Supplementary Workbook S3.1). These assessments were used to determine not only whether tools were methodologically innovative, but also the extent to which they had progressed beyond computational proof-of-concept toward experimental, preclinical, and ultimately clinical application (Supplementary Workbook S3.2).

### 2.7 Methodological quality, applicability, and risk of bias

Methodological quality, applicability, and risk of bias were assessed using a structured PROBAST+AI-informed framework adapted to the specific characteristics of AI/ML tools used in precision phage therapy (Moons et al., 2019, 2025). The adaptation retained four core domains: Domain 1, biological entities and data sources; Domain 2, predictors; Domain 3, outcomes; and Domain 4, analysis. The assessment was structured to distinguish development quality concern from evaluation risk of bias, while applicability was assessed separately for Domains 1–3; Domain 4 was treated as methodological rather than applicability-related.

For each signaling question, reviewers recorded one of six responses (Y, PY, PN, N, NI, or NA) and documented supporting evidence or justification. Domain-level judgments were subsequently categorized as Low, High, or Unclear for development quality and evaluation risk of bias, with corresponding Low/High/Unclear applicability judgments for Domains 1–3. A single negative signaling response (PN or N) did not automatically determine a high domain-level judgment; final domain-level judgments were based on the overall pattern of evidence. Unclear judgments were reserved for cases in which the available reporting was insufficient to support a more definitive assessment.

The adaptation incorporated review-specific criteria particularly relevant to computational phage research. These included assessment of data-source representativeness and provenance; appropriateness and reproducibility of predictors; biological validity and consistency of outcome labels; homology- and identity-related leakage; phylogeny-aware data splitting; circularity and benchmark dependence; class imbalance; overfitting control; independence of training, validation, and test sets; external-validation independence; appropriate performance metrics; calibration and uncertainty reporting; decision-threshold justification; and selective-prediction coverage where applicable.

In the development assessment, Domains 1–4 addressed the adequacy and representativeness of biological data, predictor definition and realism, outcome definition and labeling, and analytical methodology, respectively. The analysis domain additionally considered sample size relative to model complexity, class imbalance, overfitting, validation strategy, leakage-resistant data partitioning, hyperparameter optimization, uncertainty and calibration, and transparent model comparison. In the evaluation assessment, particular attention was given to the independence and representativeness of evaluation datasets, genuinely external validation, leakage-free partitioning, transparent performance reporting, appropriate comparators and metrics, optimistic bias, identity-, homology-, and phylogeny-aware splitting, confidence or variability estimates, and prespecified or justified decision thresholds.

The assessment of included studies was performed independently by three reviewers, with each reviewer applying the predefined signaling questions and domain-level judgment framework to the available evidence. Reviewer judgments were recorded together with the supporting rationale and unresolved information gaps. Disagreements in signaling responses or domain-level judgments were discussed among the three reviewers and resolved by consensus. The completed assessment records, including study-level signaling responses, domain judgments, and narrative rationales, were retained in Supplementary Workbook S4. The resulting framework was used to identify recurrent concerns involving data-source representativeness, predictor realism, outcome validity, data leakage, calibration, overfitting, and uncertainty reporting.

### 2.8 Data synthesis and statistical analysis

Evidence synthesis was primarily descriptive. Evidence synthesis was primarily descriptive. Geographic representation was examined descriptively by summarizing the number and proportion of studies contributed by each country/region. Because the review was designed as a heterogeneous evidence map and did not perform a quantitative meta-analysis of a common effect measure, formal statistical assessment of publication bias using methods such as funnel-plot asymmetry or Egger-type tests was not considered applicable. Instead, geographic concentration was treated as a potential source of selection and generalizability bias and was considered in the interpretation of the findings. Counts and percentages were used to summarize study selection, publication trends, geographic distribution, study type, accessibility, module distribution, architectural family, validation maturity, and methodological quality judgments. Counts and percentages were used to summarize study selection, publication trends, geographic distribution, study type, accessibility, module distribution, architectural family, validation maturity, and methodological quality judgments. Temporal patterns in architecture adoption were synthesized across publication years, and module-by-architecture distributions were examined to identify concentration of specific AI/ML families in particular functional domains. AI_PTRL values were summarized using medians and interquartile ranges. Methodological quality results were aggregated across domains to generate distributions of low, unclear, and high concern as well as overall and domain-level critical flaw burdens. Figures and tables were produced directly from the curated review dataset.

### 2.9 Reporting approach

The review was reported as a structured systematic evidence synthesis with explicit documentation of study selection and exclusion reasons. A PRISMA-style flow of records through identification, screening, eligibility, and inclusion was used in the results.

## 3 Results

### 3.1 Study selection

**Figure 1:**
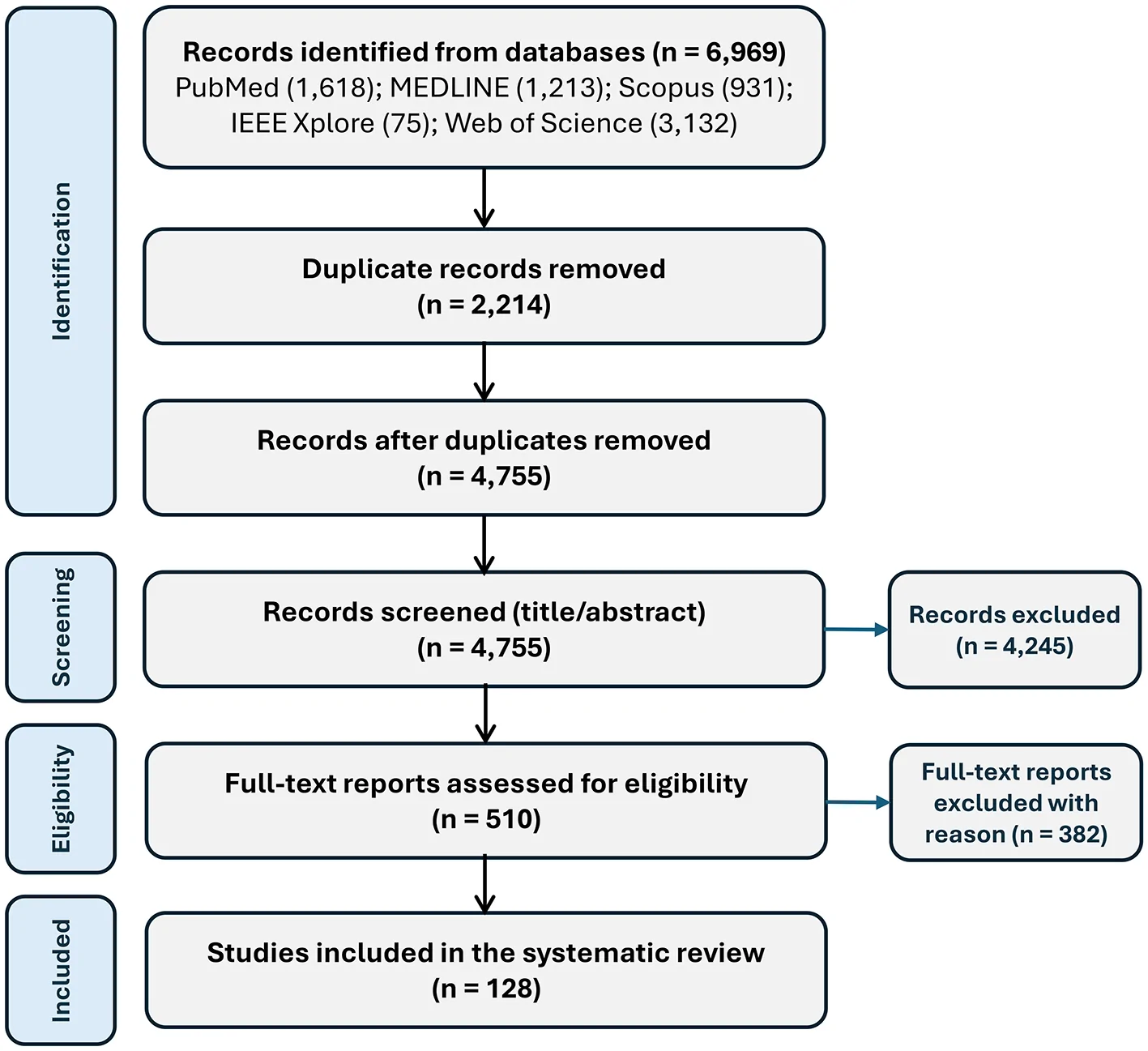
PRISMA 2020 flow diagram for study selection.

From five bibliographic databases (Web of Science, PubMed, MEDLINE, Scopus, and IEEE Xplore), we identified 6,969 records. After deduplication of 2,214 duplicates, 4,755 unique records were screened by title and abstract and excluded 4,245 records. Afterward, 510 reports were screened at the full-text level for eligibility; 382 of these reports were excluded for various reasons, and finally, a total of 128 studies were included in the final synthesis (Figure 1).

During a full text review, the most common reasons for exclusion were studies without any relevant AI/ML component (156, 40.8%); phage-related bioinformatics studies that do not contain any AI/ML (84, 22.0%); and laboratory or clinical virology studies not involving AI/ML techniques (62, 16.2%). Further exclusions included general virology studies using AI/ML (31, 8.1%), non-primary research article types such as reviews or editorials (25, 6.5%), and non-phage therapy-relevant studies (24, 6.3%; Table 1).

**Table 1:** Summary characteristics of included AI/ML tools in precision phage therapy (*n* = 128).

Characteristic	Value
Study set	
Included studies/tools	128
Unnamed tools coded (UN)	2
Temporal characteristics	
Publication years (range)	2012–2025
Publication year (median)	2023
Publications by period	2012–2015: 4 (3.1%); 2016–2019: 13 (10.2%); 2020–2021: 19 (14.8%); 2022–2023: 40 (31.25%); 2024–2025: 52 (40.63%)
Geographic contribution	
Unique countries/regions	31
Single-country studies	92 (71.87%)
Multi-country studies	36 (28.13%)
Top 10 contributing countries/regions (no. of studies)	China 59; USA 23; Hong Kong (China) 16; Australia 9; UK 8; Germany 8; Belgium 6; Denmark 6; France 5; Japan 4
Study types and accessibility	
Study class n (%)	**Method/tool development:** 115 (89.8%); **method development** **+** **benchmarking:** 6 (4.7%); **platform/database/resource:** 4 (3.1%); **decision-support/translational:** 3 (2.3%)
Validation maturity n (%)	***In silico***: 101 (78.91%); ***in silico*** **+** ***in vitro*****:** 23 (17.97%); ***in silico*** **+** ***in vivo*****:** 1 (0.78%); ***in silico*** **+** ***in vitro*** **+** ***in vivo*****:** 3 (2.34%)
Availability n (%)	***C*****:** 56 (43.75%); ***C*** **+** ***P*****:** 10 (7.81%); ***W*****:** 15 (11.72%); ***W*** **+** ***C*****:** 23 (17.97%); ***W*** **+** ***P*****:** 1 (0.78%); ***W*** **+** ***C*** **+** ***P*****:** 8 (6.25%); ***NS*****:** 15 (11.72%)
Landscape overview	
Modules distribution n (%)	***M*****1:** [phage detection and classification] 10 (7.81%); ***M*****2:** [safety and contamination screening] 4 (3.13%); ***M*****3:** [genome/protein annotation and functional inference] 33 (25.78%); ***M*****4:** [host range and interaction determinants] 43 (33.59%); ***M*****5:** [lifestyle and lysogeny] 10 (7.81%); ***M*****6:** [resistance and defense systems] 8 (6.25%); ***M*****7:** [therapeutic component discovery**] 8 (6.25%);** ***M*****8:** [therapeutic design and optimization] 7 (5.47%); ***M*****9:** [infrastructure/platforms/resources] 5 (3.91%)
Primary AI/ML paradigm n (%)	**CML:** 38 (29.69%); **DL:** 25 (19.53%); **FM:** 8 (6.25%); **GGL:** 12 (9.38%); **GM:** 1 (0.78%); **HIS:** 39 (30.47%); **PSM:** 5 (3.91%)
Maturity	
Clinical translation (AI_PTRL midpoint), median (IQR)	6 (IQR 4–6)

Percentages use *n* = 128 as denominator. Country counts attribute one study to each listed country (multi-country studies contribute to multiple countries). Validation and availability are classified exclusively using rule-based parsing of the “Validation Strategy” and “Code/Model” fields. TRL midpoint is extracted from the reported AI_PTRL range where present (e.g., TRL 2–3 -> 2.5). *Availability codes indicate the mode(s) by which each AI/ML tool can be accessed*. C denotes publicly available source code (e.g., GitHub, GitLab, Bitbucket, Zenodo); *W* denotes an accessible web-server or online interface; *P* denotes a packaged or deployable implementation (e.g., Docker/Apptainer container, Conda/PyPI package, standalone CLI/GUI). Combined codes (e.g., *W* + *C, C* + *P, W* + *C* + *P*) indicate tools providing multiple access modes. NS indicates that availability was not explicitly stated in the original publication. Bold text indicates category headings/subheadings used for grouping summary characteristics and does not denote statistical significance.

### 3.2 Summary characteristics of included AI/ML tools in precision phage therapy

A total of 128 AI/ML tools/studies were summarized in this systematic review (including 2 unnamed tools; Table 1). The included studies were published between 2012 and 2025, with a median publication year of 2023 (Table 1), suggesting that most of the activity in this field is quite recent. The selected studies surged over time, increasing from just 4 studies (3.1%) in 2012–2015 and 13 (10.2%) in 2016–2019 to 19 (14.8%) in 2020–2021, 40 (31.25%) in years 2022–2023 finally, 52 (40.63%) studies were in years 2024–2025 (Table 1). This temporal pattern reveals a significant increase in AI/ML research for precision phage therapy development in the last few years.

The included studies reveal a wide international research distribution, with contributions from 31 different countries and regions (Table 1). Most studies were conducted within single countries (92, 71.87 %), whereas 36 studies (28.13%) were conducted on a multinational basis (Table 1). By geographical distribution, China accounted for the largest number of studies (59), followed by USA (23) and Hong Kong (China) (16), with additional contributions from Australia (9), UK (8), Germany (8), Belgium (6), Denmark (6), France (5) and Japan (4; Table 1). This distribution indicates a marked geographic concentration of the evidence base, with China contributing 59 of the 128 included studies (46.1%); however, country counts were not mutually exclusive because multinational studies were attributed to each contributing country/region.

In terms of study design, this data set was predominantly comprised of method/tool development studies (115, 89.8%), with only small proportions focusing on method development with benchmarking (6, 4.7%), platform/database/resource development (4, 3.1%) and decision-support/translational applications (3, 2.3%; Table 1), suggesting that the field is primarily innovation-driven with relatively little emphasis on translational deployment or integration of clinical decision-support systems.

For validation maturity, the majority of tools were still *in silico* (Table 1). Of 128 papers, 101 (78.91%) reported only *in silico* validation (Table 1); 23 (17.97%) combined *in silico* and *in vitro* validation; 1 (0.78%) reported *in silico* plus *in vivo* validation; and just 3 studies (2.34%) incorporated all three levels: *in silico, in vitro*, and *in vivo* (Table 1).

The included studies showed variable tool accessibility (Table 1). The most frequent availability mode was public source code only (*C*) as reported for 56 tools (43.75%). Other access patterns included code and packaged implementation (*C* + *P*: 10, 7.81%), webserver (*W*: 15, 11.72%), webserver and code (*W* + *C*: 23, 17.97%), webserver and packaged implementation (*W* + *P*: 1, 0.78%) and webserver, code and packaged implementation (*W* + *C* + *P*: 8, 6.25%). However, 15 studies (11.72%) did not specify any public availability (Table 1).

**Figure 2:**
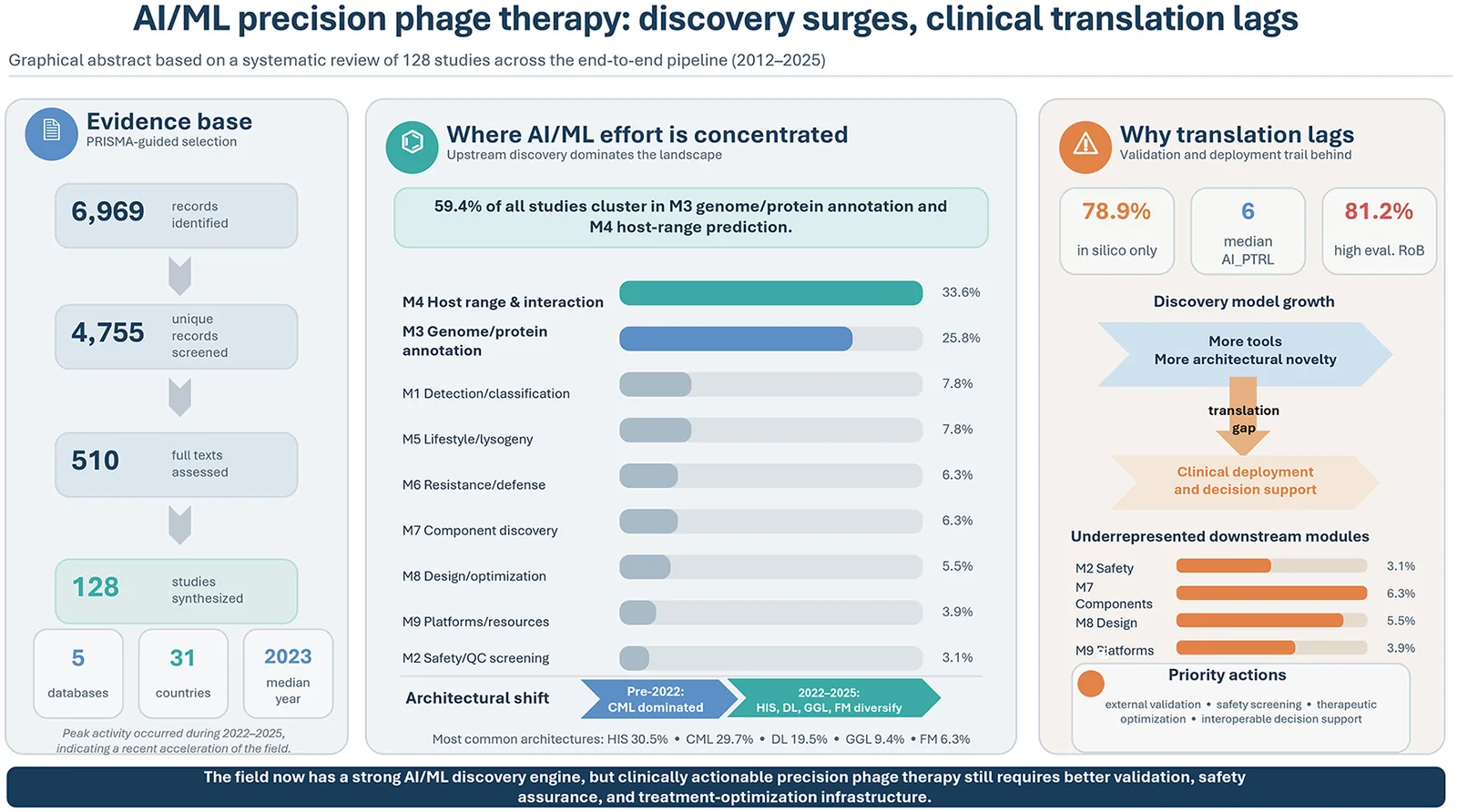
Graphical abstract of the systematic review and major findings. AI/ML, artificial intelligence/machine learning; CML, classical machine learning; DL, deep learning; FM, foundation models; GGL, graph and geometric learning; GM, generative models/design approaches; HIS, hybrid/integrated systems; PSM, probabilistic/statistical models; AI_PTRL, AI phage translational readiness level.

To structure the evidence collection process and map AI/ML tools across the end-to-end precision phage therapy pipeline, we developed a module-based classification, illustrated in Figure 2 and the Supplementary Table S1, that was used throughout the results. Tools included across the phage therapy pipeline were unevenly distributed (Table 1), and Table 2 provides a study-level mapping of each tool's module and functional category. The largest proportion of studies belonged to *M*4: host range and interaction determinants (43, 33.59%), followed by *M*3: genome/protein annotation and functional inference (33, 25.78%; Table 1). The smallest contributions were observed in *M*9: Infrastructure/Platforms/Resources (5, 3.91%); *M*8: Therapeutic Design and Optimization (7, 5.47%); and *M*2: Safety and Contamination Screening Applications (4, 3.13%; Table 1). This pattern indicates that current AI/machine learning approaches focus on phage-host matching and functional prediction, but relatively few tools focus on design, safety, and infrastructure functions.

**Table 2:** Domain–functional category mapping of AI/ML tools in precision phage therapy (*n* = 128).

Module (*M*)	Functional category (FC)	Tools included	Year range	Studies *N* (%)	Percent within module	Module total *N* (%)
*M*1	1.1 (Phage sequence identification, contig classification and recovery)	HVSeeker (Al-Najim et al., 2025), MetaPhaPred (Ma et al., 2023), PhaMer (Shang et al., 2022), PharaCon (Bai et al., 2025), Seeker (Auslander et al., 2020), VFM (Liu et al., 2019), VIBRANT (Kieft et al., 2020), VirMiner (Zheng et al., 2019), VirSearcher (Liu et al., 2023)	2019–2025	9 (7.03%)	90%	10 (7.81%)
	1.2 (Virus taxonomy/classification)	PhaGCN (Jiang et al., 2023)	2021	1 (0.78%)	10%	
*M*2	2.1 (Phage–plasmid–chromosome classification)	3CAC (Pu and Shamir, 2022), PPR-Meta (Fang et al., 2019)	2019–2022	2 (1.56%)	50%	4 (3.13%)
	2.2 (Contamination/QC screening)	vClean (Wagatsuma et al., 2025)	2025	1 (0.78%)	25%	
	2.3 (Therapeutic suitability exclusion flags)	PhageLeads (Yukgehnaish et al., 2022)	2022	1 (0.78%)	25%	
*M*3	3.1 (Protein functional annotation/functional inference)	GE-PHI (Pan et al., 2025), GOPhage (Guan et al., 2025), IBPred (Yuan et al., 2022), iPHLoc-ES (Shatabda et al., 2017), PHPred (Gao et al., 2025), MCP2T-RF/PM (Lee et al., 2022), PhagePromoter (Sampaio et al., 2019), pVOG (Grazziotin et al., 2017)	2016–2025	8 (6.25%)	24.2%	33 (25.78%)
	3.2 (Virion/structural protein prediction)	BERT-PVP (Ma et al., 2025c), DeephageTP (Chu et al., 2022), DeePVP (Fang et al., 2022), iPVP-MCV (Han et al., 2021), PBVP (Zhang et al., 2015), PVP-ML Predictor (Barman et al., 2023), PhagePred (Pan et al., 2018), PhageScanner (Albin et al., 2024), PhageTailFinder (Zhou et al., 2023), PhANNs (Cantu et al., 2020), PhaVIP (Shang et al., 2023b), Pred-BVP-Unb (Arif et al., 2020), ProtPhage (Ou et al., 2025), PVPred (Ding et al., 2014), PVP-SVM (Manavalan et al., 2018), RBPseg (Klein-Sousa et al., 2025), STEP3 (Thung et al., 2021), Un002 (Ru et al., 2019), Un003 (Tan et al., 2018), PVP Predictor (Liu et al., 2022), iVIREONS (Seguritan et al., 2012), VirionFinder (Fang and Zhou, 2021)	2012–2025	22 (17.19%)	66.7%	
	3.3 (Regulatory element prediction)	DPProm (Wang et al., 2022), iProm-phage (Shujaat et al., 2022), PhagePromoter-pipeline (Fernbach et al., 2025)	2019–2022	3 (2.34%)	9.1%	
*M*4	4.1 (Phage–host association prediction)	Bacteriophage host prediction (Boeckaerts et al., 2021), CHERRY (Shang and Sun, 2022), CL4PHI (Zhang et al., 2023), CM-PHI (Pan et al., 2026), CoMPHI (Bodaka and Kolliputi, 2025), ContigNet (Tang et al., 2022), DeepHost (Ruohan et al., 2022), DeepPBI-KG (Wei et al., 2024), DSPHI (Wei et al., 2025b), Empathi (Boulay et al., 2025b), GERMAN-PHI (Wang et al., 2023), GSPHI (Pan et al., 2023a), HoPhage (Tan et al., 2022), Host Taxon Predictor (Gačan et al., 2019), HostG (Shang and Sun, 2021), iPHoP (Roux et al., 2023), LHPre (Wang J. et al., 2025), MI-RGC (Wei et al., 2025a), MoEPH (Chen et al., 2025b), MVPHI (Coclet et al., 2024), PBIP (Ma et al., 2025a), PB-LKS (Qiu et al., 2024), PGCN (Ma et al., 2025b), PhageHostLearn (Boeckaerts et al., 2024), PHIAF (Li and Zhang, 2022), PHISGAE (Xiao et al., 2025), PHIStruct (Gonzales et al., 2025), PHP (Lu et al., 2021), PHPGAT (Liu et al., 2025), PHPGCA (Du et al., 2023), PHPRBP (Gao et al., 2025), PPI-based prediction (Camejo et al., 2025), PredPHI (Li et al., 2021), PTBGRP (Pan et al., 2023b), PHI-ML (Leite et al., 2018), ProtT5-RBP-RF (Gonzales et al., 2023), Genotype-Driven Driver Mutation Inference (Lucia-Sanz et al., 2024), VHIP (Bastien et al., 2024), vHULK (Amgarten et al., 2022), PhageTB (Aggarwal et al., 2023)	2018–2025	40 (31.25%)	93%	43 (33.59%)
	4.2 (RBP/tail fiber identification and profiling)	PhageRBPdetection (Boeckaerts et al., 2022), SpikeHunter (Yang et al., 2024)	2022–2024	2 (1.56%)	4.7%	
	4.3 (Host receptor/adsorption determinant prediction)	RBP-HostReceptor-ML. (Unnikrishnan et al., 2023)	2023	1 (0.78%)	2.3%	
*M*5	5.1 (Lifestyle classification)	BACPHLIP (Hockenberry and Wilke, 2021), DeePhafier (Miao et al., 2024), DeePhage (Wu et al., 2021), DeepPL (Zhang et al., 2024b), PHACTS (McNair et al., 2012), PhaTYP (Shang et al., 2023c), ProkBERT PhaStyle (Juhasz et al., 2025), PSOSP (Hao et al., 2025)	2012–2025	8 (6.25%)	80%	10 (7.81%)
	5.2 (Prophage identification and boundary calling)	Prophage hunter (Song et al., 2019); PhageBoost (Siren et al., 2021)	2019–2021	2 (1.56%)	20%	
*M*6	6.1 (Anti-CRISPR/anti-defense prediction)	Acr ML predictor (Gussow et al., 2020); AcRanker (Eitzinger et al., 2020); PaCRISPR (Wang et al., 2020); PreAcrs (Zhu et al., 2022); AcrNET (Li et al., 2023); AOminer (Yang et al., 2023); PACRGNN (Wang Y. et al., 2025)	2020–2025	7 (5.47%)	87.5%	8 (6.25%)
	6.2 (Bacterial defense system profiling)	Deepdefense (Hauns et al., 2024)	2024	1 (0.78%)	12.5%	
*M*7	7.1 (Endolysin/lysin discovery and prediction)	DeepLysin (Zhang et al., 2024a), DeepMineLys (Fu et al., 2024), SPAED (Boulay et al., 2025a)	2024–2025	3 (2.34%)	37.5%	8 (6.25%)
	7.2 (Depolymerase prediction)	DePP (Magill and Skvortsov, 2023); DepoScope (Concha-Eloko et al., 2024); PDP-Miner (Gauthier et al., 2025); PhageDPO (Vieira et al., 2025)	2023–2025	4 (3.13%)	50%	
	7.3 (Therapeutic payload/protein design outputs)	AlphaDesign (Jendrusch et al., 2025)	2025	1 (0.78%)	12.5%	
*M*8	8.1 (Cocktail/selection recommendation)	Predictive phage therapy (Keith et al., 2024), Predictive phage-host model (Gaborieau et al., 2024)	2024	2 (1.56%)	28.6%	7 (5.47%)
	8.2 (Dosing/PK-PD/treatment optimization)	Phage therapy parameter optimization (Plunder et al., 2022), paretoPairFinder (Plunder et al., 2024)	2022–2024	2 (1.56%)	28.6%	
	8.3 (Phage engineering and synthetic design)	megaDNA (Shao and Yan, 2024), PERPHECT (Ataee et al., 2022), prophage activation platform (Musrrat et al., 2025)	2022–2025	3 (2.34%)	42.9%	
*M*9	9.1 (Integrated platforms and web servers)	PhaBOX (Shang et al., 2023a), PhaGAA (Wu et al., 2023), PhageGE (Zhao et al., 2024), PhageScope (Wang et al., 2024)	2023–2024	4 (3.13%)	80%	5 (3.91%)
	9.2 (Databases and reference resources)	BFVD (Kim et al., 2024a)	2025	1 (0.78%)	20%	

Percentages for studies number and module total are calculated using *n* = 128. Tools are uniquely assigned to one best functional category.

In terms of methodological paradigm, the most frequent class was hybrid/integrated systems (HIS; 39, 30.47%), closely followed by classical machine learning (CML; 38, 29.69%; Table 1). Deep learning (DL) accounted for 25 studies (19.53%), graph and geometric learning (GGL) 12 (9.38%), and foundation models (FM) 8 studies (6.25%). Probabilistic/statistical models (PSM) were relatively rare (5, 3.91%), and generative models (GM) were infrequent (1, 0.78%; Table 1). Table 2 provides a description of the study-level distribution of these paradigms as well as their associated module and functional-category assignments.

Overall tool maturity was modest, with an AI_PTRL median of 6 (IQR 4–6; Table 1). This indicates that most of the included tools were classified in the mid-development stage, providing computational proof-of-concept and sometimes early biological validation, but still short of translational or clinical implementation.

### 3.3 Functional landscape of AI/ML tools across the precision phage therapy pipeline

**Figure 3:**
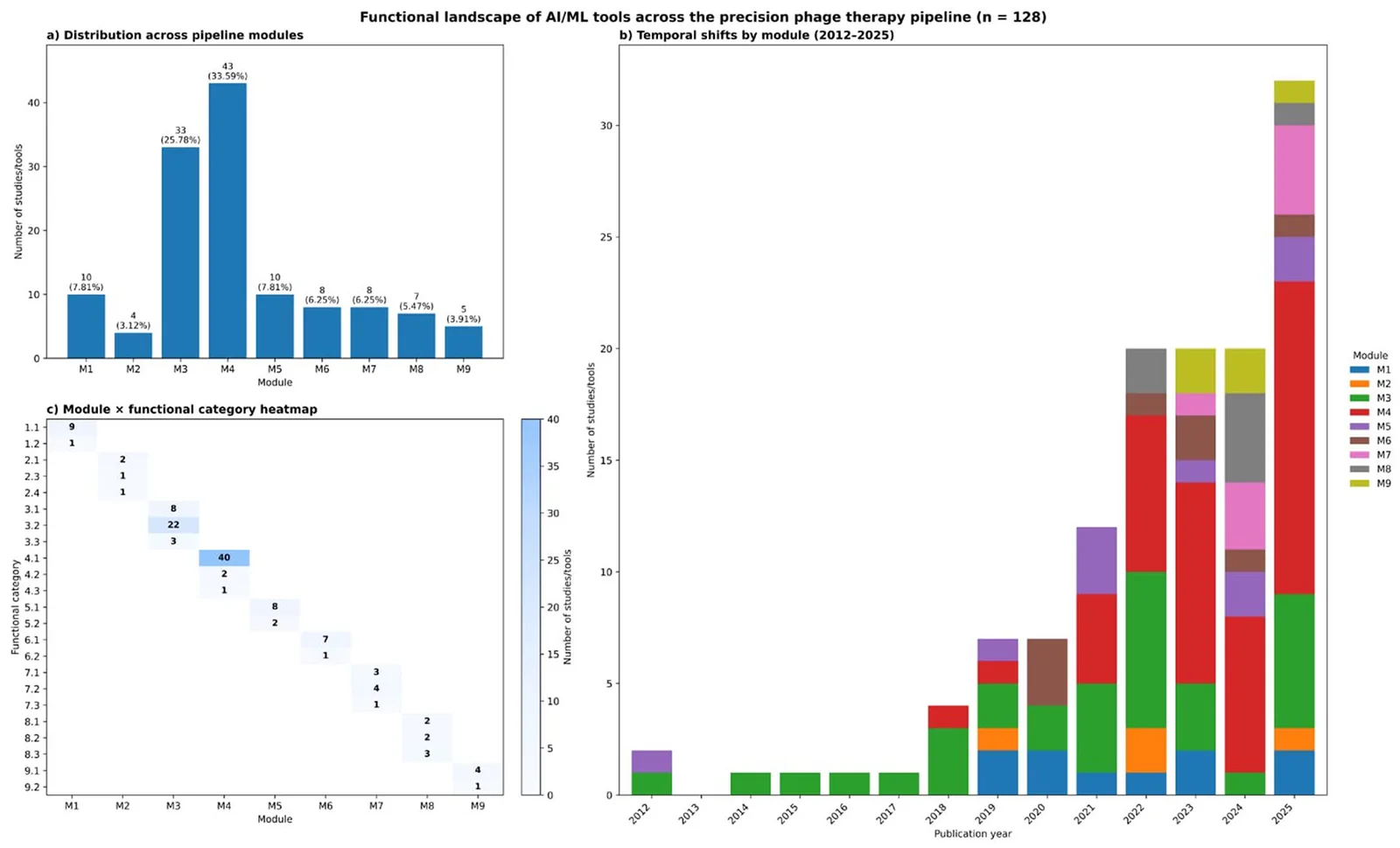
Functional landscape of AI/ML tools across the precision phage therapy pipeline (*n* = 128). **(A)** Distribution of included studies across pipeline modules (*M*1–*M*9). **(B)** Temporal shifts in research emphasis across the pipeline, illustrated by stacked bar plots of module-specific study counts by publication year (2012–2025). **(C)** Heatmap of module-by-functional-category counts summarizing the distribution of specific AI/ML tasks within each module; color intensity corresponds to the number of studies per cell. Functional categories correspond to the FC definitions in Table 2.

As shown in Figure 3A and Table 2, the included AI/ML tools were distributed unevenly across pipeline modules. *M*4 (host range and interaction determinants) was the largest module, comprising 43 studies (33.59%), followed by *M*3 (genome/protein annotation and functional inference) with 33 (25.78%). Modules *M*1 (Phage detection and classification) and *M*5 (Lifestyle and lysogeny) were each represented in 10 studies (7.81%), while *M*6 (Resistance and defense systems) and *M*7 (Therapeutic component discovery) each had 8 studies (6.25%), and *M*8 (Therapeutic design and optimization) contributed seven studies (5.47%). The least represented units were *M*9 (Infrastructure/platforms/resources), with five studies (3.91%), and *M*2, with only four studies (3.13%).

Table 2 and Figure 3B further showed a marked temporal expansion of the field. Between 2012 and 2017, no major advances were achieved beyond *M*3 (with contributions only beginning to appear sporadically for *M*5). However, starting in 2018, the pipeline had significant diversification; *M*1 and *M*2 were introduced in 2019 and *M*6 emerged by 2020. A clear acceleration was observed after 2021, followed by broad multi-module growth in 2022–2025. This late-phase expansion was driven predominantly by *M*4, which showed the most pronounced increase, alongside continued growth in *M*3 and the later emergence of *M*7–*M*9. By 2024–2025, the field had shifted from a narrow emphasis on annotation-focused tasks toward a broader, though still uneven, end-to-end precision phage therapy landscape.

Figure 3C and Table 2 demonstrated that most modules were dominated by a single principal functional category. Within *M*1, FC1.1: Phage sequence identification, contig classification and recovery accounted for 9 of 10 studies, whereas FC1.2: Virus taxonomy/classification was represented by only 1 study. In *M*3, FC3.2: Virion/structural protein prediction was the dominant category with 22 studies, compared with 8 in FC3.1: Protein functional annotation/functional inference and 3 in FC3.3: Regulatory element prediction. *M*4 was even more concentrated, with 40 of 43 studies assigned to FC4.1: Phage–host association prediction, while FC4.2: RBP/tail fiber identification and profiling and FC4.3: Host receptor/adsorption determinant prediction accounted for only two and one studies, respectively. Similar concentration patterns were observed in *M*5 (FC5.1: Lifestyle classification, eight studies), *M*6 (FC6.1: Anti-CRISPR/anti-defense prediction, seven studies), and *M*9 (FC9.1: Integrated platforms and web servers, four studies), whereas *M*7 and *M*8 were more distributed across their internal categories.

### 3.4 Architectural landscape of AI/ML methods in precision phage therapy

**Figure 4:**
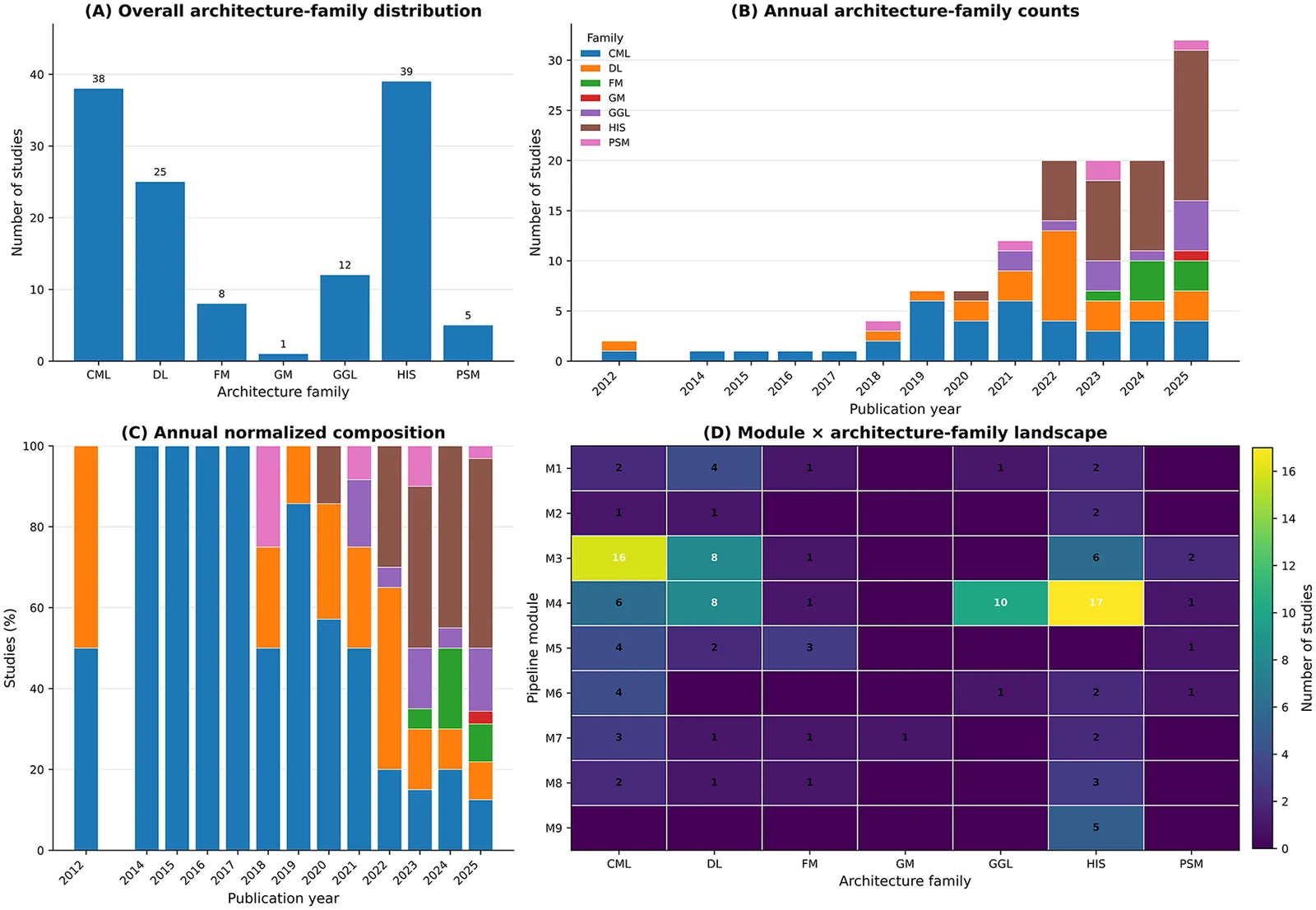
Architectural landscape of AI/ML tools across the precision phage therapy pipeline (*n* = 128). **(A)** Overall distribution of included studies across architecture families: classical machine learning (CML), deep learning (DL), foundation models (FM), generative models/design (GM), graph and geometric learning (GGL), hybrid/integrated systems (HIS), and probabilistic/statistical models (PSM). **(B)** Annual counts of studies by architecture family across the study timeline (2012–2025). **(C)** Annual normalized composition showing the relative contribution of each architecture family per year. **(D)** Heatmap of module-by-architecture-family counts across pipeline modules (*M*1–*M*9), with color intensity indicating the number of studies in each cell.

As noted in Figures 4A, B, the trends observed reflect an increase not only in the quantity of phage therapy research but also its diversity. Before 2022, classical machine learning (CML) was dominant with 23 of 36 studies (63.9%); deep learning (DL) accounted for 8 studies (22.2%). Other models, such as hybrid/integrated systems (HIS), were seldom implemented. A marked increase in architectural diversity was observed between 2022 and 2025, with hybrid/integrated systems (HIS) being the most prominent at 38/92 (41.3%), followed by deep learning (DL) at 17/92 (18.5%), CML at 15/92 (16.3%), graph and geometric learning (GGL) at 10/92 (10.9%), foundational models (FM) at 8/92 (8.7%), probabilistic/statistical models (PSM) (3/92, 3.3%) and generative models (GM) (1/92, 1.1%). FM: emerged after 2023, while pure generative models (GM) appeared in 2025. This suggests that the growth in the field correlates with not only an increase in publications, but also with the advancement of quality and diversity of approaches exhibiting improvements of computational tools for phage therapy. Figures 4A, B implies a methodological progression from standalone predictors to integrative systems able to incorporate multiple data types, representations or modeling paradigms.

Figure 4C further illustrates the changing relative composition of architectural families over time, showing the increasing contribution of HIS, DL, GGL, and FM during the later study period. Figure 4D demonstrates that architectural diversification did not take place uniformly throughout the precision phage therapy pipeline. *M*4 had the widest and densest architectural distribution with 17 HIS, 10 GGL, 8 DL and 6 CML studies in addition to lone FM and PSM entries, suggesting that host-related prediction has become the main proving ground for advanced/multimodal architectures. In contrast, *M*3 was focused on CML (16 studies) and DL (8 studies) but also contained a smaller HIS component (6 studies). Other modules had more particularistic architectural signatures: *M*9 was comprised solely of HIS, *M*5 was driven by CML and FM, and *M*1 was led by DL with minor contributions from CML, HIS, GGL, and FM. In sum, Figure 4D shows that there is strong coupling between functional role and methodological family across models, although the greatest concentration of architectural experimentation occurs in phage–host interactions prediction.

#### 3.4.1 Spatiotemporal distribution of architectural families across pipeline modules

**Figure 5:**
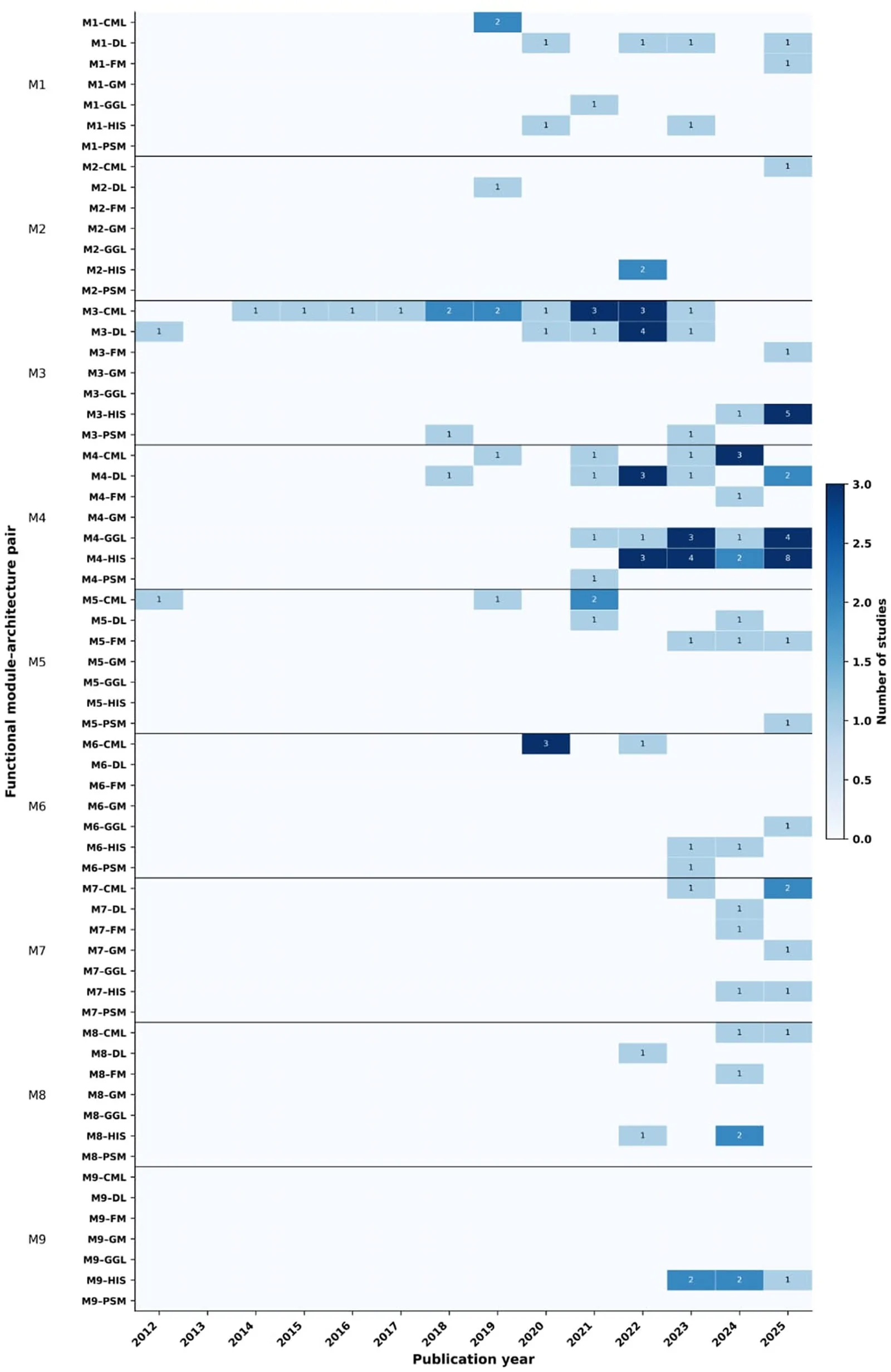
Spatiotemporal distribution of AI/ML architectural families across functional modules in precision phage therapy (2012–2025; *n* = 128). Heatmap showing annual study counts stratified by combined functional module (*M*1–*M*9) and primary AI/ML architectural family (CML, DL, FM, GM, GGL, HIS, PSM). Rows correspond to module–architecture pairs, while columns represent publication years. Color intensity reflects the number of studies per cell.

Figure 5 showed that pre-2020 AI-assisted phage therapy research was primarily centered on annotation-oriented applications with narrow functional modules scope and architectural technology. *M*3 dominated functional modules from 2012 to 2017, with isolated early signals from *M*5, and classical machine learning (CML) was the dominant machine learning architecture used, apart from a single early deep-learning contribution in *M*3. In 2018–2019, this pattern began to broaden, with the emergence of probabilistic/statistical modeling in *M*3, DL in *M*4, and CML-based tools in *M*1 and *M*4.

However, between 2020 and 2022, the machine learning architecture starts to evolve, becoming more intensive and widespread. In 2020 CML-based tools emerged in *M*6, while *M*1 included both DL and HIS. By 2021 *M*4 evolved to include CML, DL, GGL, and PSM; while *M*5 remained largely focused on CML. The major shift in 2022 was that *M*3—DL became widespread (four studies), and *M*4 included three DL-based tools and three HIS-based tools with one tool using GGL architecture.

The major spatiotemporal expansion occurred in 2023–2025 and was driven primarily by *M*4. In 2023, *M*4-HIS and GGL were four and three studies, respectively, establishing interaction-determinant modules as the principal site of architectural diversification. This intensified further in 2025, when eight *M*4-HIS and four *M*4-GGLbased tools, while *M*3-HIS also rose to five studies. In parallel, later-stage modules became more visible: *M*9 tools were entirely HIS architecture-based, *M*7 used CML, FM, HIS, and a GGL architectures, and *M*8 combined CML, DL, FM, and HIS across optimization-oriented tasks.

### 3.5 Validation maturity and translational readiness across the precision phage therapy pipeline

**Figure 6:**
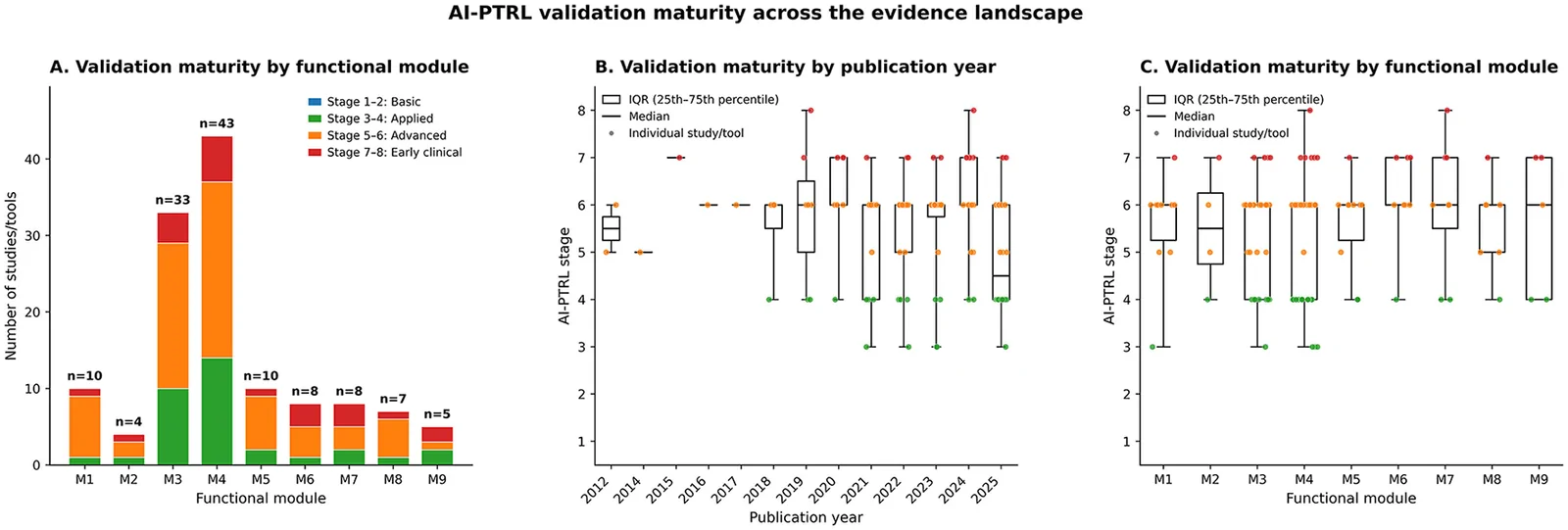
Validation maturity and AI/ML translational readiness (AI_PTRL) across the precision phage therapy landscape (*n* = 128). **(A)** Validation maturity by functional module (*M*1–*M*9). Stacked bar chart showing the number of studies/tools in each module, stratified into four mutually exclusive evidence categories: *in silico* only, *in silico* + *in vitro, in silico* + *in vivo*, and *in silico* + *in vitro* + *in vivo*. The total number of studies/tools in each module is indicated above the corresponding bar. Upstream discovery-oriented modules are predominantly supported by computational evidence, whereas therapy-adjacent modules show comparatively greater experimental substantiation. **(B)** AI_PTRL by publication year (2012–2025). Boxplots summarize the distribution of AI_PTRL values for each publication year. The box represents the interquartile range (IQR; 25th−75th percentiles), the horizontal line represents the median, and individual points represent individual studies/tools. Point colors correspond to the four validation-maturity categories defined in panel A. **(C)** AI_PTRL by functional module. Boxplots show the distribution of AI_PTRL values within each module; the box represents the IQR (25th−75th percentiles), the horizontal line represents the median, and individual studies/tools are shown as colored points with horizontal jitter to improve visibility of overlapping observations. Higher AI_PTRL values indicate greater estimated translational maturity.

Validation maturity was predominantly computational across the precision phage therapy landscape, although substantial differences were observed among functional modules (Figure 6A). Modules *M*1 and *M*9 were entirely supported by *in silico*-only validation, whereas *M*3 was also predominantly computational, with 27 of 33 studies/tools (81.8%) classified as *in silico* only. In contrast, several therapy-adjacent modules showed greater incorporation of experimental evidence. *M*7 exhibited the greatest diversity of validation evidence, with four of eight studies/tools (50.0%) classified as *in silico* only, two (25.0%) incorporating *in vitro* evidence, and two (25.0%) incorporating both *in vitro* and *in vivo* evidence. In *M*4, 8 of 43 studies/tools (18.6%) incorporated *in vitro* experimental validation. *M*5 and *M*8 similarly included studies extending beyond purely computational evaluation, although computational-only evidence remained predominant. These findings indicate that experimental validation is beginning to emerge in downstream and therapy-adjacent areas but remains substantially less common than computational validation across the landscape.

Translational readiness showed a less pronounced progression than the expansion of methodological activity (Figure 6B). AI_PTRL values remained concentrated around intermediate preclinical stages, with annual median values generally near 5.5–6.0 during most years with sufficient observations. Earlier studies (2012–2019) were relatively sparse and heterogeneous, whereas publication volume increased substantially after 2021 without a corresponding sustained upward shift in median AI_PTRL. The annual median reached 6.0 in 2022, 2023, and 2024 but decreased to 4.5 in 2025. Thus, the increasing volume of AI/ML applications was not accompanied by a consistent increase in estimated translational readiness, with most studies remaining below the highest stages of translational deployment.

At the module level, AI_PTRL distributions were generally centered around intermediate stages, with most modules showing median values of approximately 5.5–6.0 (Figure 6C). *M*2 showed the lowest median at 5.5, whereas the remaining modules were centered at a median of 6.0. *M*4 and *M*7 exhibited the broadest observed ranges, with studies/tools reaching AI_PTRL stage 8, indicating that these modules contained applications reaching the highest observed level of estimated translational readiness in the dataset. In contrast, *M*5 and *M*6 showed relatively tighter distributions around intermediate AI_PTRL stages, suggesting more consistent but predominantly preclinical maturity. The module-level analysis indicates that although some therapy-adjacent areas included applications reaching higher levels of estimated translational readiness, the overall landscape remained concentrated at intermediate stages of development.

### 3.6 Assessment of methodological quality, risk of bias, and applicability

**Figure 7:**
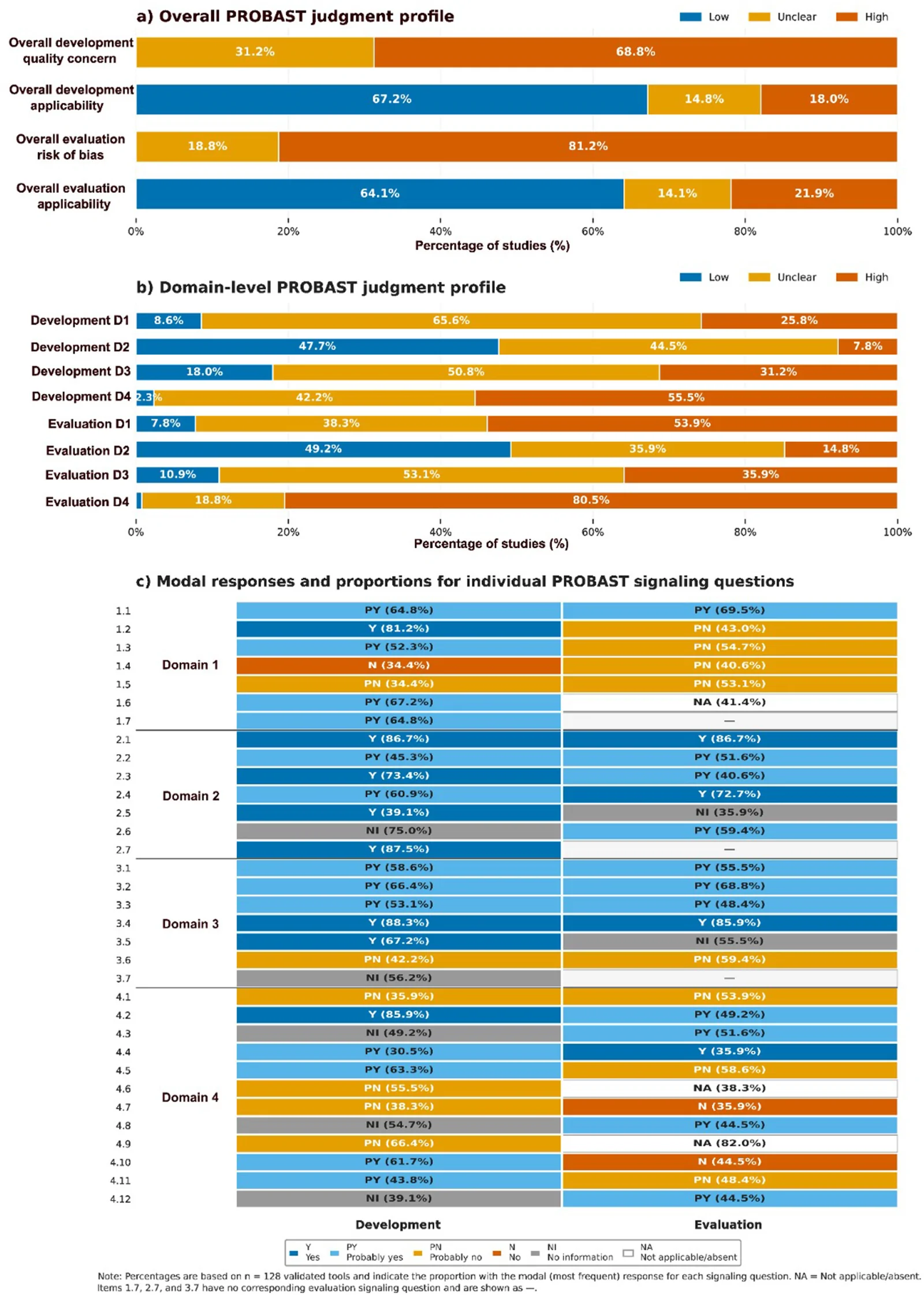
Adapted PROBAST profile of included AI/ML tools/models (*n* = 128). **(A)** Overall judgments for development quality concern, evaluation risk of bias, and applicability. **(B)** Domain-level distributions of development quality concern and evaluation risk of bias across the four adapted PROBAST domains. Domain 1, Participants and data sources; Domain 2, Predictors and predictor handling; Domain 3, Outcome definition and assessment; and Domain 4, Analysis and model evaluation. **(C)** Item-level distribution of responses to individual adapted PROBAST signaling questions, shown separately for development and evaluation. Y, yes; PY, probably yes; PN, probably no; N, no; NI, no information; NA, not applicable/absent. “NI” indicates that the available information was insufficient to determine whether the signaling question was satisfied. In panels A and B, **Low**, low concern/risk; Unclear, unclear concern/risk; and High, high concern/risk. Percentages represent the proportion of included tools/models (*n* = 128). The heatmap in panel C presents the modal response and corresponding proportion for each signaling question. PROBAST, Prediction model Risk Of Bias ASsessment Tool; AI, artificial intelligence; ML, machine learning.

Risk of bias was predominantly high across the 128 included tools/models, particularly at the evaluation stage. Overall, 104 of the 128 studies (81.2%) were judged to have high evaluation risk of bias, while the remaining 24 (18.8%) were classified as unclear; development quality concern was also frequently high, with 88 studies (68.8%) rated as having high concern and 40 (31.2%) classified as unclear (Figure 7A). By contrast, applicability concerns were comparatively more favorable, with low concern observed in 86 studies/tools (67.2%) for applicability in the development setting and in 82 (64.1%) for applicability in the evaluation setting (Figure 7A). At the domain level, Domain 4 showed the least favorable profile, with high concern in 71 studies (55.5%) during development and high risk of bias in 103 (80.5%) during evaluation, indicating that the analysis and model evaluation domain represented the principal source of methodological concern (Figure 7B). In contrast, Domain 2 showed the most favorable profile for development quality concern and evaluation risk of bias, with the highest proportions of low-concern judgments for development (61, 47.7%) and low-risk judgments for evaluation (63, 49.2%; Figure 7B). Applicability was also highly favorable overall, with low concern observed in 119 studies (93.0%) for development and 118 (92.2%) for evaluation (Figure 7A). Domains 1 and 3 showed mixed patterns, characterized by substantial proportions of unclear judgments alongside notable high-concern and high-risk ratings (Figure 7B). Examination of the individual signaling questions further indicated that unfavorable judgments were associated mainly with issues concerning the representativeness and description of data sources (Domain 1), the consistency and real-world applicability of predictor definitions and handling (Domain 2), the definition, consistency, and biological relevance of outcome measures (Domain 3), and, most prominently, analytical and validation practices, including effective sample size, missing-data handling, overfitting control, leakage-resistant data partitioning, model selection, and completeness of performance assessment (Figure 7C).

## 4 Discussion

This study serves as a systematic review of artificial intelligence and machine learning (AI/ML) tools relevant to precision phage therapy. This systematic review reveals that the AI/ML landscape in precision phage therapy is defined by a profound discovery-translation imbalance. While the field has built a powerful computational engine for upstream discovery, this has not been matched by comparable development of the downstream decision-support systems required for clinical deployment, creating a significant translational bottleneck.

The geographic distribution of the evidence base warrants consideration when interpreting the overall findings. China contributed 59 of the 128 included studies (46.1%), representing the largest national contribution in the review, although multinational studies were attributed to each contributing country/region. This concentration may partly reflect the substantial research activity in AI/ML-enabled phage biology within China, but it may also introduce regional imbalance into the evidence base. Geographic clustering can influence the apparent prominence of particular research priorities, methodological approaches, and functional applications if research outputs, datasets, or publication practices differ across regions. We therefore interpret the observed dominance of host-range prediction, genome/protein annotation, and related computational applications as representing the retrieved international evidence base rather than necessarily a globally uniform research landscape. A formal statistical assessment of publication bias was not performed because the review was an evidence-mapping synthesis of heterogeneous study designs, models, outcomes, and performance measures without a common effect estimate suitable for conventional publication-bias tests. Nevertheless, the geographic concentration is recognized as a potential source of selection and generalizability bias and should be considered when extrapolating the review findings to regions that are less represented in the literature.

The systematic mapping of 128 AI/ML tools reveals a field characterized by explosive growth but asymmetrical development, heavily skewed toward the initial stages of the precision phage therapy pipeline. This first section of our discussion interprets this core finding, analyzing the implications of a research landscape where 59.37% of all efforts are concentrated in just two upstream modules: Genome/protein annotation and functional inference (*M*3; 25.78%) and Host Range and Interaction Determinants (*M*4; 33.59%).

This concentration is both a reflection of historical necessity and a potential bottleneck for clinical translation. The predominance of *M*3 and *M*4 tools logically addresses the most immediate and data-rich challenges in phage biology: deciphering the function of genes within the vast “dark matter” of phage genomes and predicting phage-bacterial interactions, which bacterial host. The success of deep learning models like DeePVP for virion protein prediction and transformer-based tools like PhaTYP for lifestyle classification exemplifies how modern AI excels at extracting patterns from genomic sequences, tasks that are foundational to any rational therapy development pipeline (Fang et al., 2022; Shang et al., 2023c). Similarly, the surge in *M*4 tools, particularly those employing graph neural networks (e.g., CHERRY) and ensemble methods, represents a concerted effort to replace the slow, empirical process of laboratory host range testing with rapid computational prediction (Shang and Sun, 2022). This shift is crucial for accelerating the initial matchmaking between a patient's bacterial isolate and potential therapeutic phages (phage-host matching). However, this upstream skew reveals a significant strategic gap. The comparative underrepresentation of downstream modules—particularly Resistance and defense systems (*M*6; 6.25%); Therapeutic Design & Dosing (*M*8; 5.47%), and Therapeutic Component Discovery (*M*7; 6.25%). This concluded that the AI/ML community has prioritized building a powerful “discovery engine” while the essential “clinical steering system” remains underdeveloped. This imbalance is important because many of the challenges addressed by these downstream modules—including resistance management, therapeutic optimization, pharmacological considerations, safety, and treatment design—are closely linked to the clinical translation and regulatory development of phage-based therapeutics (Turner et al., 2024). For instance, while a tool may accurately predict a phage-host match, the absence of robust AI for screening genomic contaminants (*M*2) or for optimizing synergistic cocktail regimens and pharmacokinetic dosing (*M*8) leaves clinicians without actionable guidance for safe and effective deployment (Doud et al., 2025). The very few tools in *M*8, such as PERPHECT, which employs Pareto optimization for cocktail design, highlight the nascent stage of this vital research direction (Keith et al., 2024).

This distribution suggests that phage therapy ML tools evolution has been primarily driven by the availability of data (e.g., genomic sequences) and the tractability of specific prediction tasks, rather than by a pipeline-balanced roadmap addressing all translational hurdles. The consequence is a maturation gradient where computational discovery is highly advanced, but its integration into a complete, clinically actionable workflow is incomplete. Bridging this gap requires a conscious rebalancing of research focus toward the complex, multi-factorial problems of therapeutic optimization, safety assurance, and regimen personalization, which are inherently harder but essential for moving from *in silico* prediction to bedside application (Doud et al., 2025).

A defining characteristic of the AI/ML landscape in precision phage therapy is its rapid and decisive architectural evolution. Our analysis demonstrates a clear temporal shift from classical, feature-engineered models toward modern paradigms centered on representation learning, a transition that underscores the field's alignment with the broader AI revolution in the life sciences. One of the clearest patterns in this review is that the AI/ML landscape in precision phage therapy has undergone a genuine methodological transition rather than simple numerical growth. Data analysis showed that older studies were dominated by classical machine learning, whereas the recent expansion phase is increasingly shaped by hybrid systems, deep learning, graph-based learning, and foundation-model-style approaches. Collectively, these newer and more integrative paradigms displaced the earlier CML-centered landscape, indicating that precision phage therapy has entered a more diverse architectural era (Yi et al., 2022). This transition mirrors broader trends across bioinformatics and biomedicine, where the field has progressively moved from hand-crafted feature engineering toward deep representation learning, graph-based modeling, and large pretrained models that can exploit high-dimensional biological data more effectively (Min et al., 2017).

The shift from predominantly classical machine-learning approaches toward more diverse architectures, including deep learning, hybrid/integrated systems, graph and geometric learning, and foundation models, is also biologically plausible. Early phage AI/ML studies often addressed comparatively constrained prediction problems, such as lifestyle classification or sequence-based annotation, where curated features and conventional classifiers were often sufficient. By contrast, newer questions in precision phage therapy increasingly involve higher-order biological complexity, including phage–host association prediction, receptor-binding protein discovery, anti-defense prediction, and therapeutic design. These tasks are more compatible with architectures that can integrate heterogeneous inputs, capture non-linear sequence-function relationships, and explicitly represent relational structure. This helps explain why *M*4 emerged as the main center of architectural experimentation in dataset, with 17 HIS, 10 GGL, 8 DL, and 6 CML studies. More broadly, this pattern is consistent with the wider biomedical AI literature, where graph representation learning has been highlighted as especially suitable for systems defined by interacting biological entities, and where foundation-model approaches are increasingly valued for transfer learning across sparse downstream tasks (Li et al., 2022).

At the same time, greater architectural sophistication should not be mistaken for translational maturity. Despite the rise of HIS, DL, GGL, and FM, validation in this review remained overwhelmingly computational, with 78.91% of the studies were restricted to *in silico* evidence and an overall AI_PTRL median of 6 (IQR 4–6). This suggests that the field is advancing faster in model sophistication than in real-world validation. That imbalance is not unique to phage research: across biomedical AI, recent methodological analyses have repeatedly shown that impressive benchmark performance often outpaces external validation, deployment realism, and clinically meaningful integration (Chen et al., 2025a; Doud et al., 2025). In phage therapy specifically, recent perspectives likewise argue that predictive tools may accelerate isolate-specific matching and design, but that the field still faces major translational bottlenecks, including limited efficacy-grade clinical evidence, standardization challenges, and the persistent difficulty of moving from promising computational tools to reliable therapeutic deployment (Doud et al., 2025; Sokol et al., 2025).

A further implication is that future progress should focus not only on architectural novelty, but also on methodological robustness. This is particularly important in biological machine learning, where small, imbalanced, and highly structured datasets increase vulnerability to overfitting, leakage, and inflated performance estimates (Bernett et al., 2024). Recent methodological guidance has emphasized that biological ML pipelines are especially prone to hidden data leakage, while broader reviews of small-data machine learning in molecular science have highlighted the difficulty of building stable and generalizable models under limited sample conditions (Ultsch and Lötsch, 2024). These concerns are highly relevant to phage datasets, where host-range labels, sequence similarity, taxonomic clustering, and uneven organism coverage can all compromise apparent generalization if not handled rigorously. Accordingly, the next phase of precision phage therapy AI should prioritize stronger external validation, leakage-aware benchmarking, and experimental confirmation, so that architectural advances translate into clinically credible decision support rather than remaining confined to upstream computational discovery (Bernett et al., 2024).

The present review showed a predominance of unfavorable methodological judgments, with high concern for development quality and an even greater concentration of high risk of bias at the evaluation stage, while applicability was comparatively more favorable. This overall pattern is consistent with the broader PROBAST literature, in which machine-learning prediction model studies are frequently judged to be at high overall risk of bias and the analysis domain is repeatedly identified as the principal source of methodological weakness (Dhiman et al., 2022; Langenhuijsen et al., 2023a). Large cross-specialty and topic-specific reviews have reported similarly high proportions of unfavorable judgments, and a meta-review of PROBAST-based systematic reviews likewise found that high risk of bias remains common across prediction research, especially in the analysis domain (Navarro et al., 2021; Dhiman et al., 2022).

The particularly unfavorable profile observed in Domain 4 is methodologically plausible in the context of phage AI/ML research. Phage prediction studies often rely on datasets with uneven representation of phage and bacterial diversity, incomplete experimentally characterized interactions, and heterogeneous biological contexts. In phage–host prediction, these limitations are compounded by fragmented or incomplete phage genomes, uneven representation of bacterial hosts in reference databases, and the difficulty of defining infection relationships using simple binary labels (Coclet and Roux, 2021). Recent benchmarking of computational host-range prediction tools further showed that performance varies with the taxonomic level of prediction, with strain-level prediction remaining substantially more challenging than species- or genus-level prediction, and highlighted limitations in standardized evaluation and experimental validation of reference interactions (Howell et al., 2024). These characteristics can make independent data partitioning, control of overfitting, and reliable assessment of model performance and generalizability particularly challenging in phage prediction studies. Such field-specific constraints provide a plausible explanation for the high frequency of Domain 4 concerns observed in the present review. Similar methodological weaknesses have been documented more broadly in biomedical prediction modeling. In a systematic review of supervised machine-learning prediction models, Andaur Navarro and colleagues found that the analysis domain was most frequently rated at high risk of bias; 56% of models had an inadequate number of events per candidate predictor, 41% handled missing data inadequately, and 39% assessed overfitting improperly (Navarro et al., 2021). Similarly, an oncology review identified analysis-related methodological problems as a major contributor to high risk of bias (Dhiman et al., 2022).

By contrast, the more favorable applicability profile suggests that many included tools were broadly aligned with their intended prediction context even when their methodological quality was limited (Moons et al., 2019). This distinction is central to PROBAST and has been retained in PROBAST + AI: applicability addresses whether the participants, predictors, and outcomes match the review question, whereas risk of bias and quality concern address whether the model was developed and evaluated in a trustworthy way (Moons et al., 2025). Accordingly, low applicability concern should not be interpreted as evidence of robustness or readiness for use; rather, it indicates that many studies were asking the “right” question but answering it with methods that remain vulnerable to bias (Wolff et al., 2019; de Jong et al., 2021).

In the context of AI/ML tools for phage research, these findings imply that methodological limitations currently lie less in the biological relevance of the prediction targets and more in the rigor of model development and, especially, validation. Reported performance estimates should therefore be interpreted cautiously, as they may be optimistic when based on limited internal validation, incomplete handling of missingness, or inadequate safeguards against overfitting and data leakage (Moons et al., 2019, 2025; Navarro et al., 2021). Future work in this field should prioritize stronger external and independent validation, explicit leakage control, fuller reporting of calibration and other clinically or biologically meaningful performance measures, and closer adherence to AI-specific methodological and reporting guidance such as PROBAST + AI and TRIPOD + AI (Dhiman et al., 2022; Doud et al., 2025).

A further point worth acknowledging is that PROBAST-based assessments themselves are not entirely free of subjectivity. The 2023 meta-review found low inter-rater agreement across several PROBAST domains and signaling questions, together with a ceiling effect in which many studies cluster at high risk of bias. This does not invalidate the present findings, but it supports interpreting fine-grained differences between domains or studies with some caution and reinforces the value of using structured, transparent, and explicitly documented assessment procedures (Langenhuijsen et al., 2023b; Moons et al., 2025). A related limitation is the uneven number of studies/tools represented across functional modules and AI/ML architecture families. Several categories contained fewer than ten studies, limiting the stability and interpretability of comparisons involving sparsely represented architectures or modules. Subgroup comparisons based on small or highly unbalanced numbers of studies generally have limited statistical power, and their findings should therefore be interpreted cautiously (Cuijpers et al., 2021). In addition, estimates based on small evidence bases may be disproportionately influenced by individual studies. Accordingly, comparisons involving sparsely represented architectures should be regarded as descriptive rather than as evidence of systematic differences in methodological performance or translational maturity. This limitation is particularly relevant when interpreting apparent differences in AI_PTRL or quality profiles across architecture families, where the number of contributing studies varies substantially.

A further limitation concerns the temporal restriction of the literature search to 2012–2025. The year 2012 was selected to capture an early period of explicit AI/ML application to phage-related sequence analysis, while maintaining a focused scope aligned with the development of AI/ML methods relevant to precision phage therapy. However, computational and statistical approaches to phage biology predated 2012, and some of these approaches may have provided conceptual or methodological foundations for subsequent AI/ML applications. Although the present review specifically excluded conventional computational/bioinformatics studies without an explicit AI/ML component, as defined in the eligibility criteria, the temporal restriction may nevertheless have resulted in omission of some earlier precursor or AI/ML-related studies. Therefore, the findings should be interpreted as reflecting the AI/ML evidence base within the predefined 2012–2025 timeframe rather than the complete historical development of computational approaches in phage research.

A related limitation is the uneven geographic representation of the included literature. The predominance of studies from China, with smaller contributions from many other regions, means that the evidence base may reflect regional research priorities, computational resources, dataset availability, and publication practices. Although this geographic concentration does not by itself demonstrate regional publication bias, it may limit the extent to which the observed methodological and functional patterns can be generalized to the global AI/ML phage research landscape.

## 5 Conclusions

To conclude, this systematic review shows that AI and ML have become fast-growing components of precision phage therapy, with 128 tools and studies identified through December 2025. Most of today's progress are focused on upstream applications, especially genome and protein annotation and host prediction. In this regard, computational approaches have greatly accelerated the pace and scale of phage characterization. Yet, to date there has not been a corresponding translational progress that mirrors this methodological advancement. While *in silico* validation remains the most widespread application of ID, downstream fields essential for clinical implementation, including safety screening, therapeutic optimization and decision support are still in their infancy. These findings imply that, for this field, the next stage should represent not only greater algorithmic sophistication but also stronger external validation, clinically relevant benchmarking, interoperable data infrastructure, and greater interdisciplinary collaboration. The priority should therefore be to translate computational promise into stable, reproducible, and clinically actionable evidence capable of supporting precision phage therapy for multidrug-resistant infections.

## Data Availability

The original contributions presented in the study are included in the article/supplementary material, further inquiries can be directed to the corresponding author.
