# Supplementary material for: Precision phage therapy in the AI/ML era: a systematic review of discovery-to-clinical translation evidence
Source: Front Microbiol. 2026 Sep 9;17:1873220. doi: 10.3389/fmicb.2026.1873220 (PMC13599804; doi:10.3389/fmicb.2026.1873220)
Supplement: Supplementary file 1 [file Data_Sheet_1.zip › Supplementary/Supplementary Table S2.docx]

**Supplementary Table S2.1 Search framework, objective, information sources, and core Boolean structure**

| **Component** | **Description** |
| --- | --- |
| **Objective and scope** | A systematic search was performed to identify AI/ML-enabled computational tools or models relevant to the precision phage therapy pipeline, spanning upstream discovery and annotation through interaction inference and therapeutic design/optimization. Searches targeted primary research describing tools, algorithms, or models applied to phage therapy-relevant endpoints. |
| **Information sources** | Web of Science; PubMed; MEDLINE; Scopus; IEEE Xplore |
| **Time span** | 2012–2025 |
| **Search structure** | All databases used the same three-block Boolean structure: Block 1 AND Block 2 AND Block 3 |
| **Block 1: Phage therapy / phage context** | phage therapy OR bacteriophage therapy OR phage treatment OR bacteriophage treatment OR phage OR bacteriophage |
| **Block 2: AI/ML and computational methods/tools** | artificial intelligence OR AI OR machine learning OR deep learning OR neural network OR computational biology OR bioinformatics OR in silico OR software OR web OR tool OR algorithm OR natural language processing OR NLP |
| **Block 3: Precision phage therapy tasks (pipeline terms)** | host prediction OR phage-host interaction OR lifestyle prediction OR genome design OR lysin OR depolymerase OR phage genome OR metagenomic* OR phage engineering OR synthetic biology OR cocktail |
| **Implementation notes** | Where supported, terms were searched in title, abstract, and keyword fields; otherwise, database-default all-fields searching was used. Truncation was applied where available, for example metagenomic*. |

**Supplementary Table S2.2 Database-specific search strings used for literature retrieval**

| **Database** | **Search field / interface** | **Database-specific query string** | **Notes** |
| --- | --- | --- | --- |
| **Web of Science** | Topic field (TS) | TS=((phage therapy OR bacteriophage therapy OR phage treatment OR bacteriophage treatment OR phage OR bacteriophage) AND (artificial intelligence OR AI OR machine learning OR deep learning OR neural network OR computational biology OR bioinformatics OR in silico OR software OR web OR tool OR algorithm OR natural language processing OR NLP) AND (host prediction OR phage-host interaction OR lifestyle prediction OR genome design OR lysin OR depolymerase OR phage genome OR metagenomic* OR phage engineering OR synthetic biology OR cocktail)) | Topic search includes title, abstract, author keywords, and Keywords Plus. |
| **Scopus** | TITLE-ABS-KEY | TITLE-ABS-KEY((phage therapy OR bacteriophage therapy OR phage treatment OR bacteriophage treatment OR phage OR bacteriophage) AND (artificial intelligence OR AI OR machine learning OR deep learning OR neural network OR computational biology OR bioinformatics OR in silico OR software OR web OR tool OR algorithm OR natural language processing OR NLP) AND (host prediction OR phage-host interaction OR lifestyle prediction OR genome design OR lysin OR depolymerase OR phage genome OR metagenomic* OR phage engineering OR synthetic biology OR cocktail)) | Searches title, abstract, and keywords. |
| **PubMed** | Title/Abstract | (("phage therapy"[Title/Abstract] OR "bacteriophage therapy"[Title/Abstract] OR "phage treatment"[Title/Abstract] OR "bacteriophage treatment"[Title/Abstract] OR phage[Title/Abstract] OR bacteriophage[Title/Abstract]) AND ("artificial intelligence"[Title/Abstract] OR AI[Title/Abstract] OR "machine learning"[Title/Abstract] OR "deep learning"[Title/Abstract] OR "neural network"[Title/Abstract] OR "computational biology"[Title/Abstract] OR bioinformatics[Title/Abstract] OR "in silico"[Title/Abstract] OR software[Title/Abstract] OR web[Title/Abstract] OR tool[Title/Abstract] OR algorithm[Title/Abstract] OR "natural language processing"[Title/Abstract] OR NLP[Title/Abstract]) AND ("host prediction"[Title/Abstract] OR "phage-host interaction"[Title/Abstract] OR "lifestyle prediction"[Title/Abstract] OR "genome design"[Title/Abstract] OR lysin[Title/Abstract] OR depolymerase[Title/Abstract] OR "phage genome"[Title/Abstract] OR metagenomic*[Title/Abstract] OR "phage engineering"[Title/Abstract] OR "synthetic biology"[Title/Abstract] OR cocktail[Title/Abstract])) | This format is safer than tagging the whole block once. |
| **MEDLINE** | Platform-dependent; title/abstract/keyword where available | ((phage therapy OR bacteriophage therapy OR phage treatment OR bacteriophage treatment OR phage OR bacteriophage) AND (artificial intelligence OR AI OR machine learning OR deep learning OR neural network OR computational biology OR bioinformatics OR in silico OR software OR web OR tool OR algorithm OR natural language processing OR NLP) AND (host prediction OR phage-host interaction OR lifestyle prediction OR genome design OR lysin OR depolymerase OR phage genome OR metagenomic* OR phage engineering OR synthetic biology OR cocktail)) | For Ovid MEDLINE, the same logic may be adapted using field tags such as .ti,ab,kw. |
| **IEEE Xplore** | All Metadata | ("phage therapy" OR "bacteriophage therapy" OR "phage treatment" OR "bacteriophage treatment" OR phage OR bacteriophage) AND ("artificial intelligence" OR AI OR "machine learning" OR "deep learning" OR "neural network" OR "computational biology" OR bioinformatics OR "in silico" OR software OR web OR tool OR algorithm OR "natural language processing" OR NLP) AND ("host prediction" OR "phage-host interaction" OR "lifestyle prediction" OR "genome design" OR lysin OR depolymerase OR "phage genome" OR metagenomic* OR "phage engineering" OR "synthetic biology" OR cocktail) | Use All Metadata; Abstract can be used for narrower retrieval if needed. |
