# Supplementary material for: Precision phage therapy in the AI/ML era: a systematic review of discovery-to-clinical translation evidence
Source: Front Microbiol. 2026 Sep 9;17:1873220. doi: 10.3389/fmicb.2026.1873220 (PMC13599804; doi:10.3389/fmicb.2026.1873220)
Supplement: Supplementary file 1 [file Data_Sheet_1.zip › Supplementary/Supplementary Table S1.docx]

**Supplementary Table S1. End-to-end functional taxonomy of AI/ML tools in the precision phage therapy pipeline, with module assignments, functional categories, and intended use statements.**

| **Module Code** | **Module Name** | **FC Code** | **Functional Category Name** | **Intended Use Statement** |
| --- | --- | --- | --- | --- |
| M1 | Phage / virus detection & classification | 1.1 | Phage sequence identification, contig classification & recovery | Identify and recover phage/viral sequences from contigs, assemblies, or metagenomic data to distinguish phage-derived from non-phage sequences for downstream analysis, annotation, and candidate discovery. |
| M1 | Phage / virus detection & classification | 1.2 | Virus taxonomy / classification | Assign phage/viral sequences to taxonomic groups (for example family, genus, or species) from genome- or sequence-derived features for classification, comparative analysis, and reference placement. |
| M2 | Safety & contamination screening | 2.1 | Phage–plasmid–chromosome classification | Classify assembled sequences or contigs as phage, plasmid, or chromosomal origin from sequence-derived features to reduce misclassification and improve interpretation of candidate therapeutic genomes. |
| M2 | Safety & contamination screening | 2.2 | Contamination / QC screening | Detect contamination, low-quality inputs, technical artifacts, or other quality-control failures in phage-related sequence data to support reliable downstream modeling, annotation, and therapeutic assessment. |
| M2 | Safety & contamination screening | 2.3 | Therapeutic suitability exclusion flags | Flag phage candidates that meet predefined exclusion criteria from genomic, proteomic, or annotation-derived features in order to remove candidates with therapy-relevant safety or suitability concerns from further consideration. |
| M3 | Genome / protein annotation & functional inference | 3.1 | Protein functional annotation / functional inference | Predict protein function or functional role from phage protein or gene sequence-derived features to improve annotation and support biological interpretation beyond homology-only approaches. |
| M3 | Genome / protein annotation & functional inference | 3.2 | Virion / structural protein prediction | Identify virion-associated or structural proteins from phage protein sequences or derived representations to support capsid/tail annotation, particle biology interpretation, and therapeutic component profiling. |
| M3 | Genome / protein annotation & functional inference | 3.3 | Regulatory element prediction | Predict regulatory elements, promoters, motifs, or regulatory regions from phage genomic sequence in order to support gene-expression analysis, genome interpretation, and engineering design. |
| M4 | Host range & interaction determinants | 4.1 | Phage–host association prediction | Predict the likely bacterial host taxon, strain association, or phage–host interaction likelihood from phage genomic, proteomic, or receptor-binding features to support host-range inference, matching, and therapeutic selection. |
| M4 | Host range & interaction determinants | 4.2 | RBP/tail fiber identification & profiling | Identify or characterize receptor-binding proteins, tail fibers, spikes, or adsorption-associated modules from phage protein or genome features to infer host specificity and adsorption-related function. |
| M4 | Host range & interaction determinants | 4.3 | Host receptor / adsorption determinant prediction | Predict bacterial receptors or adsorption determinants relevant to phage binding and entry from host-, phage-, or interaction-derived features to support mechanistic understanding of host specificity and treatment matching. |
| M5 | Lifestyle & lysogeny | 5.1 | Lifestyle classification | Classify phages as lytic, temperate, or related lifestyle states from genome- or protein-derived features to support therapeutic suitability screening and candidate prioritization. |
| M5 | Lifestyle & lysogeny | 5.2 | Prophage identification & boundary calling | Detect prophage regions and define prophage boundaries within bacterial genomes from sequence and genomic-context features to support genome interpretation, contamination control, and lysogeny-related assessment. |
| M6 | Resistance & defense systems | 6.1 | Anti-CRISPR / anti-defense prediction | Predict anti-CRISPR or other anti-defense proteins from phage protein or genomic features to identify mechanisms that may modulate bacterial immunity and influence therapeutic performance. |
| M6 | Resistance & defense systems | 6.2 | Bacterial defense system profiling | Identify or profile bacterial defense systems from host genomic features to characterize resistance mechanisms relevant to phage susceptibility, treatment planning, and combination design. |
| M7 | Therapeutic component discovery | 7.1 | Endolysin / lysin discovery & prediction | Predict endolysins, lysins, or related lytic enzymes from protein or genome-derived features to support enzyme-therapy candidate discovery and therapeutic component prioritization. |
| M7 | Therapeutic component discovery | 7.2 | Depolymerase prediction | Predict depolymerase proteins from phage protein or genome-derived features to identify candidates for capsule or EPS degradation, antivirulence use, and host-access enhancement. |
| M7 | Therapeutic component discovery | 7.3 | Therapeutic payload / protein design outputs | Generate, optimize, or prioritize therapeutic proteins or payloads from sequence, structure, or learned representations to support phage-based intervention design and engineered therapeutic development. |
| M8 | Therapeutic design & optimization | 8.1 | Cocktail/selection recommendation | Recommend individual phages or phage cocktail compositions for a defined host, pathogen, or infection context using predicted efficacy, compatibility, or coverage features to support therapeutic selection. |
| M8 | Therapeutic design & optimization | 8.2 | Dosing / PK-PD / treatment optimization | Optimize dosing, timing, scheduling, or PK/PD-informed treatment strategies from experimental, simulated, or patient-context data to support phage treatment planning and therapeutic control. |
| M8 | Therapeutic design & optimization | 8.3 | Phage engineering & synthetic design | Design or optimize phage genomes, modules, or synthetic constructs from sequence, structural, or systems-level features to improve therapeutic properties, host targeting, or functional performance. |
| M9 | Infrastructure/platforms/resources | 9.1 | Integrated platforms & web servers | Provide an integrated computational platform that combines one or more phage-analysis prediction or interpretation tasks to support end-to-end analysis, candidate evaluation, and user-facing decision support. |
| M9 | Infrastructure/platforms/resources | 9.2 | Databases & reference resources | Provide a curated reference resource that organizes phage-related biological, functional, or benchmarking information to support annotation, model development, comparative evaluation, and evidence retrieval. |
